# Great Salt Lake microbiology: a historical perspective

**DOI:** 10.1007/s10123-018-0008-z

**Published:** 2018-06-04

**Authors:** Bonnie K. Baxter

**Affiliations:** Great Salt Lake Institute, Westminster College, 1840 South 1300 East, Salt Lake City, UT 84105 USA

**Keywords:** Great Salt Lake, Extremophiles, Halophiles, Hypersaline, History of science

## Abstract

Over geologic time, the water in the Bonneville basin has risen and  fallen, most dramatically as freshwater Lake Bonneville lost enormous volume 15,000–13,000 years ago and became the modern day Great Salt Lake. It is likely that paleo-humans lived along the shores of this body of water as it shrunk to the present margins, and native peoples inhabited the surrounding desert and wetlands in recent times. Nineteenth century Euro-American explorers and pioneers described the geology, geography, and flora and fauna of Great Salt Lake, but their work attracted white settlers to Utah, who changed the lake immeasurably. Human intervention in the 1950s created two large sub-ecosystems, bisected by a railroad causeway. The north arm approaches ten times the salinity of sea water, while the south arm salinity is a meager four times that of the oceans. Great Salt Lake was historically referred to as sterile, leading to the nickname “America’s Dead Sea.” However, the salty brine is teaming with life, even in the hypersaline north arm. In fact, scientists have known that this lake contains a diversity of microscopic lifeforms for more than 100 years. This essay will explore the stories of the people who observed and researched the salty microbiology of Great Salt Lake, whose discoveries demonstrated the presence of bacteria, archaea, algae, and protozoa that thrive in this lake. These scientists documented the lake’s microbiology as the lake changed, with input from human waste and the creation of impounded areas. Modern work on the microbiology of Great Salt Lake has added molecular approaches and illuminated the community structures in various regions, and fungi and viruses have now been described. The exploration of Great Salt Lake by scientists describing these tiny inhabitants of the brine illuminate the larger terminal lake with its many facets, anthropomorphic challenges, and ever-changing shorelines.

## Introduction

**Fig. 1:**
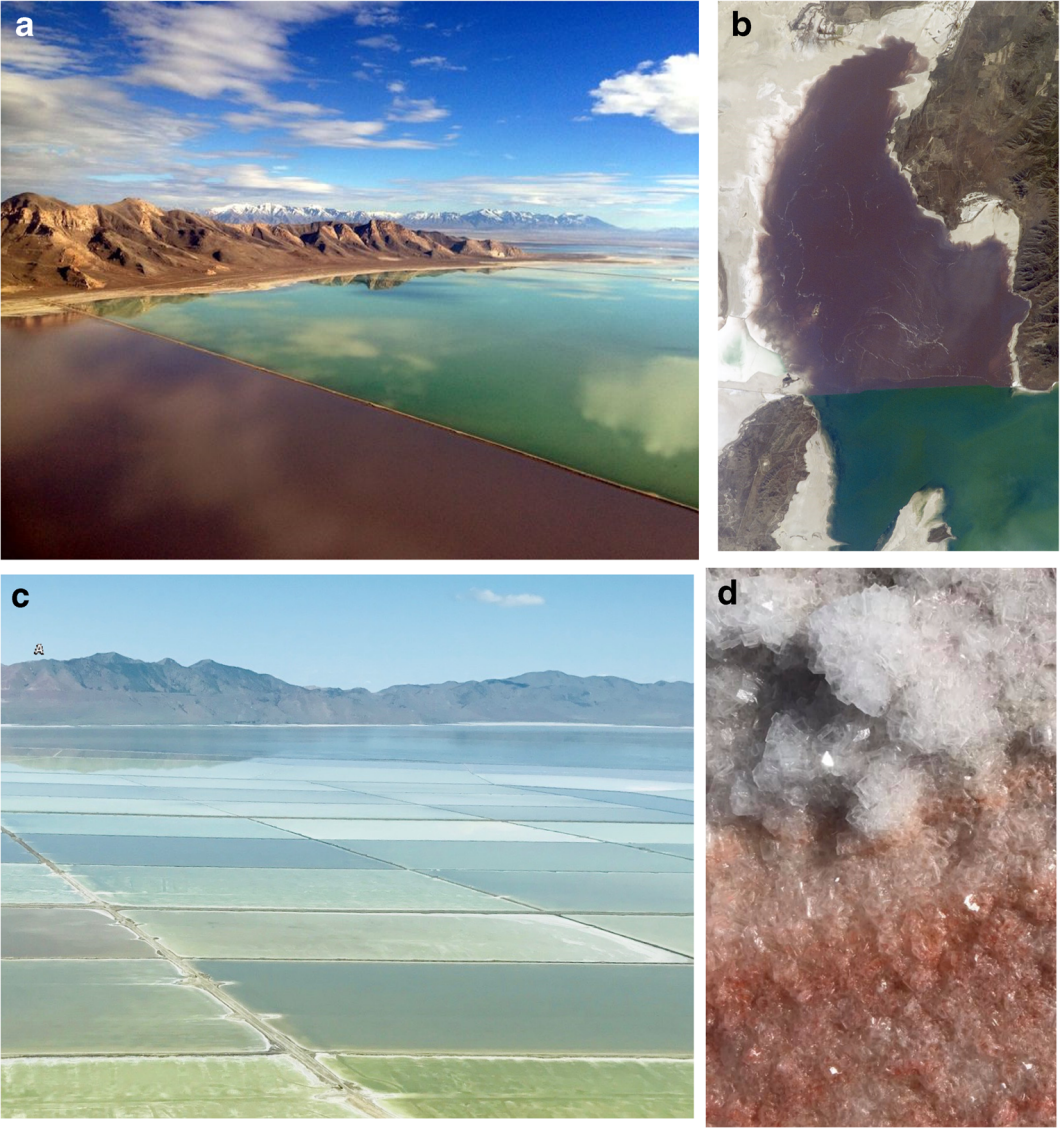
The visual coloration of Great Salt Lake by microorganisms. **a** The north arm of Great Salt Lake is a rich pink color due largely to the halophilic archaea that inhabit the hypersaline water, while the south arm is green in color, due to the rich diversity of algae species. The two arms are separated by a railroad causeway seen here. (*image credit: Patrick Wiggins*). **b** Astronauts can see the different microbial communities from space (*image credit: International Space Station, NASA, 2015*). **c** The salt ponds of Compass Minerals in spring 2016 (*image credit: Jaimi K. Butler*). **d** Carotenoids of halophilc archaea color the shoreline salt crust (*image credit: Jaimi K. Butler)*

An aerial view of Great Salt Lake (GSL) rewards the observer with a watercolor palette of hues, varying spatially and seasonally from pink to purple to orange in the north arm, and from blue to green in the south arm (Fig. 1a–c). From the shorelines, one can see that salt crusts and sandy beaches are also tinted (Fig. 1d). The coloration is due to the microbiology of the lake’s waters as tiny organisms contain carotenoid or chlorophyll pigments. This painted landscape that varies as you circumnavigate the lake underscores that a microscope is not required to see communities of microorganisms. To observe a single, individual cell, one needs a lens, but the presence of GSL's complex microbial foundation is obvious even to the casual viewer. The smell of the lake is often remarked upon, and this too is a sensory experience that informs us about the microorganisms and the work they do, decomposing and turning over nutrients in the lake brine and sediment.

If GSL is teaming with life, the richness of which is obvious, then how did it earn the nickname, “America’s Dead Sea” (The Deseret News 1907; Scientific American 1861)? Did the humans, living along the lakeshore since the formation of the largest lake in the western US, notice the microbiology of the lake? Did they harvest pink salt, use the brine for curing meat, or attempt to eat the algal mats? Did early explorers complain about the smell of the water they studied? This paper traces these observations and the microbiological studies of this unique and understudied ecosystem, in a historical context.

## Natural history of Great Salt Lake

### Paleo Lake Bonneville and the formation of Great Salt Lake

**Fig. 2:**
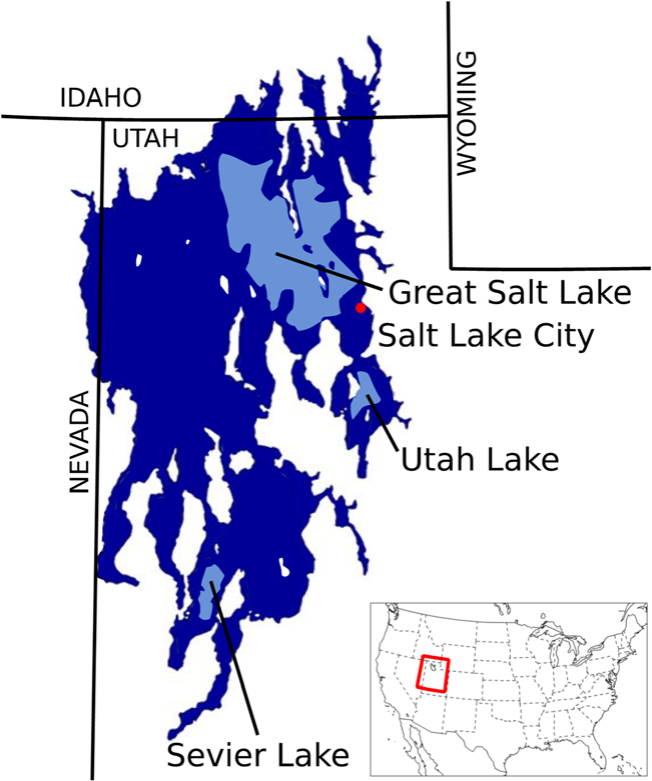
A map of Great Salt Lake in the context of state lines and the outline of Paleo Lake Bonneville shown in dark blue (Atwood et al. 2016). (image credit: Great Salt Lake Institute)

GSL sits in the Bonneville Basin, which is one of the lowest depressions in the Great Basin, the largest contiguous inland watershed in North America (Cohenour and Thompson 1966). Over time, this Basin primarily held shallow lakes such as GSL, or mudflats and playa, likely over the last several million years (Atwood et al. 2016). However, it was home to several deep lakes over the last 780,000 years, including late Pleistocene Lake Bonneville, 30–12,000 years ago, covering about 20,000 mile^2^ of western Utah and extended into eastern Nevada and southern Idaho (Oviatt et al. 1999; Shroder et al. 2016) (Fig. 2).

The transition of Lake Bonneville to GSL occurred over just a few thousand years in several stages (Atwood et al. 2016; Shroder et al. 2016). As the last ice age thawed and the Earth warmed, the water evaporated and leaked out, resulting in the current GSL lake margins about 13,000 years ago (Fig. 2). Modern GSL is the largest lake in the western United States, the fourth largest terminal saline lake in the world and the second saltiest lake on Earth next to the Dead Sea (Keck and Hassibe 1979). Captain Howard Stansbury led the earliest mapping expedition that captured the expanse of the lake in the nineteenth century (Stansbury 1855). The salinity from Stansbury’s time until now fluctuated and varies across the various regions of the lake, from that of the ocean, around 3% salt, to 34% in the salt-saturated north arm [e.g., Baxter et al. 2005; Jones et al. 2009; Naftz et al. 2011; United States Geologic Survey 2018].

### The modern Great Salt Lake ecosystem

**Fig. 3:**
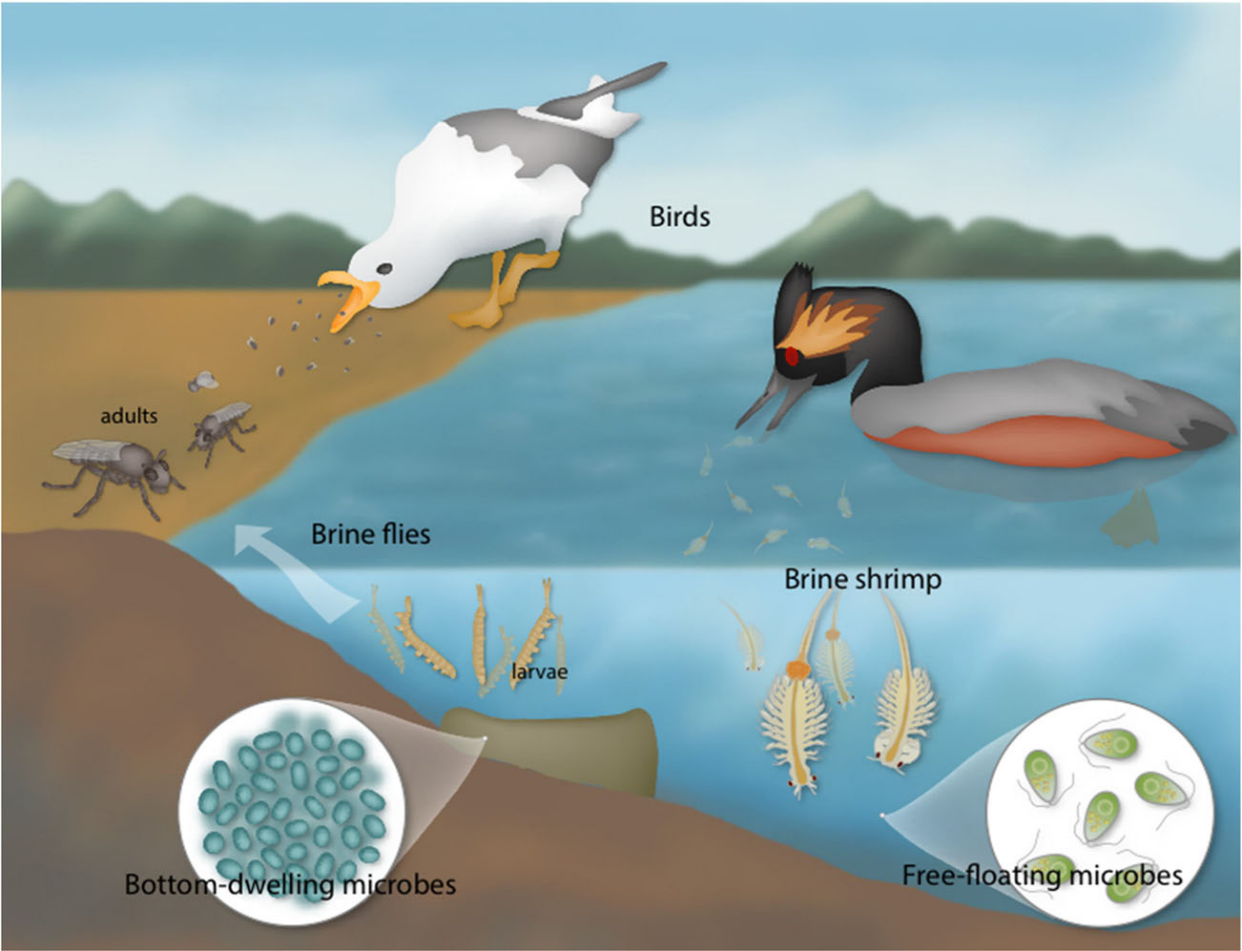
The food web of modern Great Salt Lake. In a seemingly simple food web, birds who visit the lake eat brine shrimp and brine flies, and the fly larva and shrimp eat the algae and other microorganisms in the brine. The complexity lies in the microbial activities including photosynthesis by pelagic and microbialite autotrophs and the benthic microbial processes that turnover nutrients in the ecosystem (image credit, by permission: The Genetics Science Learning Center, University of Utah)

Today’s GSL serves as a critical stop on the Pacific Flyway, hosting about ten million waterbirds (around 250 species) that spend at least part of the year here, making the lake the most important shorebird site in North America (Aldrich and Paul 2002; Bellrose 1980; Neill et al. 2016; Oring et al. 2000; Paul and Manning 2016). The immense number of birds is sustained by two invertebrate species: (1) brine shrimp (*Artemia franciscana*), which spend all phases of their life cycle in the water column, and (2) brine flies (*Ephydra* spp.), whose egg, larval and pupal stages occur in the brine (Aldrich 1912; Collins 1980; Packard Jr 1871; Roberts 2013; Verrill 1869; Wurtsbaugh and Gliwicz 2001). The microorganisms, especially algae, of GSL feed these invertebrates [e.g., Barnes and Wurtsbaugh 2015; Belovsky et al. 2011; Collins 1980; Felix and Rushforth 1979; Larson and Belovsky 2013; Roberts 2013; Wurtsbaugh and Gliwicz 2001]. The food web is simple from the macro viewpoint: birds eat shrimp and flies. However, the microbiology drives the complex biochemistry of photosynthesis, nutrient turnover, and decomposition (Fig. 3). Primary producers include pelagic phytoplankton (Larson and Belovsky 2013) and also those in microbial mats which form microbialites on the lake bottom (Chidsey Jr et al. 2015; Lindsay et al. 2017). While shrimp primarily dine on the free-floating microbes, brine fly larvae pupate on the microbialites and eat the associated cyanobacteria (Wurtsbaugh et al. 2011).

GSL is a terminal lake, which means water flows into it and not out of it. With no outlet, GSL has cycles of drought and flooding, dependent on temperature, evaporation and precipitation cycles, which makes it highly susceptible to climate change (Wurtsbaugh et al. 2017). Abundant precipitation in contributing watersheds caused the lake to rise four meters from 1983 to 1987 (Stephens 1990), and years of recent drought set a new historic low in 2016 (United States Geologic Survey 2018), the lowest elevation since 1963 (Stephens 1990). As the lake rises and falls, the salinity of the GSL water column is impacted as high precipitation can dilute the brine, and desiccating conditions can concentrate it. Therefore, salinity in the lake varies inversely with lake level, and salinity can influence the species of microorganisms present at any given time (Almeida-Dalmet et al. 2015; Boyd et al. 2014; Meuser et al. 2013).

## Humans and Great Salt Lake, insights and impacts on microbiology

### The early peoples of Utah and their interactions with Great Salt Lake

Humans have been in North America, and possibly Utah, since the Pleistocene, between 20,000 and 15,000 years ago, the time of the high point of Lake Bonneville (Madsen 2015; Madsen 2016; Raghavan et al. 2015). Though no direct evidence exists regarding their interaction with this large lake, human artifacts have been studied with respect to the rivers and wetlands in the Lake Bonneville basin (Oviatt et al. 2003). Lake Bonneville and its watershed would have given humans a food source of freshwater fish. As the water level oscillated over time, responding to a changing climate, humans would have moved to follow the changing shorelines (Madsen 1999). Archaeologists have found artifacts from Paleoamerican culture in the Great Basin area from around 14,500 to 14,300 years ago (consistent with the Provo level of receding Lake Bonneville) but none precisely around the shores of the evaporating lake [Reviewed in Madsen 2016]. As the water evaporated over a couple thousand years, and the lake got salter, human interactions with the lake would have changed.

Even as the margins of GSL were formed, ample remaining groundwater sources for freshwater springs existed for several thousand years, until the sources were significantly depleted about 9500 years ago (Madsen 2015; Oviatt et al. 2015; Simms, 2016). This geology is consistent with anthropological evidence of humans who lived in the wetlands of GSL, hunting, fishing and foraging [e.g., Madsen 1989; Madsen and Kirkman 1988]. It is very likely that salt was collected, used, and traded, given that its value is well-documented in other cultures, but because salt dissolves in rain water, physical evidence is difficult to obtain. There is evidence of occupied caves, in particular “Danger Cave” in the desert on the west side of the lake, which was occupied for likely thousands of years [Reviewed in Madsen 2014]. Perhaps a testament to the significance of the GSL wetlands to the native people, the Fremont buried dozens of their dead in this region during their occupancy along the Bear River and around the wetlands of GSL circa AD 400–1000 (Coltrain and Leavitt 2002; Parr et al. 1996; Simms, 2016).

### Recent American Indian inhabitants along the lakeshore

About 1000 years ago until the late nineteenth century, a remarkable group of Shoshonean-Paiute peoples with shared dialects occupied the Great Basin and exemplified a simple existence in a desolate, desert ecosystem (Cuch 2000; Simms, 2016). The Shoshone and Utes lived around the north part of GSL, and the Goshutes lived around the south end of the lake, extending southwest of that area. Much has been written about, in particular, the Goshute people, who are known for their deep understanding and connection to climate and cycles that influence the fauna and flora of their desert homeland. Given this, it is very likely that they utilized GSL for salt collection, for trade or self-use, and may have observed algae blooms or other microbiological processes. In fact, the language (and its dialects) of the Shoshonean peoples had a word for algae (Flowers 1957). Friction arose with the white people moving into the area from the 1847 Mormon emigration to the Overland Stagecoach and the Pony Express (Cuch 2000). The dry, salty land had more demand as Euro-Americans began farming and raising livestock. Conflict eventually resulted in attacks on the Goshutes from local militias and the US Army, killing many and ending in a forced treaty signing in 1863 that caused the people to move away from the Great Salt Lake Desert (Cuch 2000; Defa 1994).

### White traders, settlers and pioneers

From the mid-1800s forward, given the power structure in place, the people who had a relationship with GSL were largely of European descent. Journaling was routine as they needed to make detailed maps and notes regarding animals and other potential resources to share with their funders. Therefore, we have the first written, detailed accounts of the GSL during this time.

Exploration for fur trading brought white men to the west, and in 1824 they were in the area of GSL. Jedidiah Smith sent explorer, John Weber, along the Bear River and into Cache Valley, but not as far as the Bear’s terminus where it flowed into GSL (Miller 1969). Peter Skene Ogden, also a trapper, came further south, but did not report seeing GSL in his journal. Later that same year, Jim Bridger floated down the Bear River and claimed the first discovery of GSL, which he concluded, likely upon tasting the water, that it was a part of the Pacific Ocean. But Bridger’s claim was contested, as French-Canadian trapper, Etienne Provost, beat him to GSL by a few months. Given that the area was already inhabited, neither discovered GSL; that honor goes to the indigenous peoples (Cuch 2000).

In 1843, John C. Frémont, an explorer, entered GSL from the Bear River after traveling from Missouri. He led a party intent on mapping the region, describing new terrain, and doing scientific investigations (Frémont 1845; Miller 1948). Their studies mapped the topography of GSL and its islands and presented the first scientific investigation of mineral content and biology of its waters, including brine shrimp and flies (Frémont 1845). Frémont recorded stories of indigenous people eating brine fly larvae, but he did not include any notes on the microbiology per se. They collected brine for evaporation and made salt for consumption, noting its impurities, perhaps due to the microorganisms they had unwittingly sampled. Frémont did record observations of microbial activities as he wrote about the smell as they entered the brackish waters on the edge of GSL.

Westward expansion benefitted from the geographical information that resulted from these explorations and those of the trapping industry as trails were blazed and documented. The infamous “Hastings cutoff,” a departure from the Oregon trail which led through “inhospitable” land of the Goshutes to California, brought the doomed Donner-Reed party through the salt playa surrounding GSL in 1846 (Rarick 2008). Due to the hydrogen sulfide released by the sulfate-reducing microorganisms here [e.g., Boyd et al. 2017; Brandt and Ingvorsen 1997], not only was this briny muck to slow down the party, but it likely had a sulfurous smell (Bagley 2010; Rarick 2008).

In contrast, GSL was seen in a more positive light by pioneers entering Utah from the Church of Jesus Christ of Latter-Day Saints in 1847, traveling with their leader, Brigham Young. The “Mormons” immediately noted the parallel geography of the Salt Lake Valley and the Holy Land in the Middle East, each with salt and freshwater lakes joined by a river. This was, to some, affirmation that Utah was a place for their “chosen people,” just as the Holy Land was seen as the promised land for the "Lord’s people" in another time (The Church of Jesus Christ of Latter-Day Saints 1997). Some of the first group of Mormon pioneers wasted little time in heading out to swim in the briny lake waters, just 3 days after entering the valley (Hunter 1943). “We cannot sink in this water. We roll and float on the surface like a dry log. I think the Salt Lake is one of the wonders of the world,” journaled Orson Pratt, a member of Young’s initial pioneer company.

**Fig. 4:**
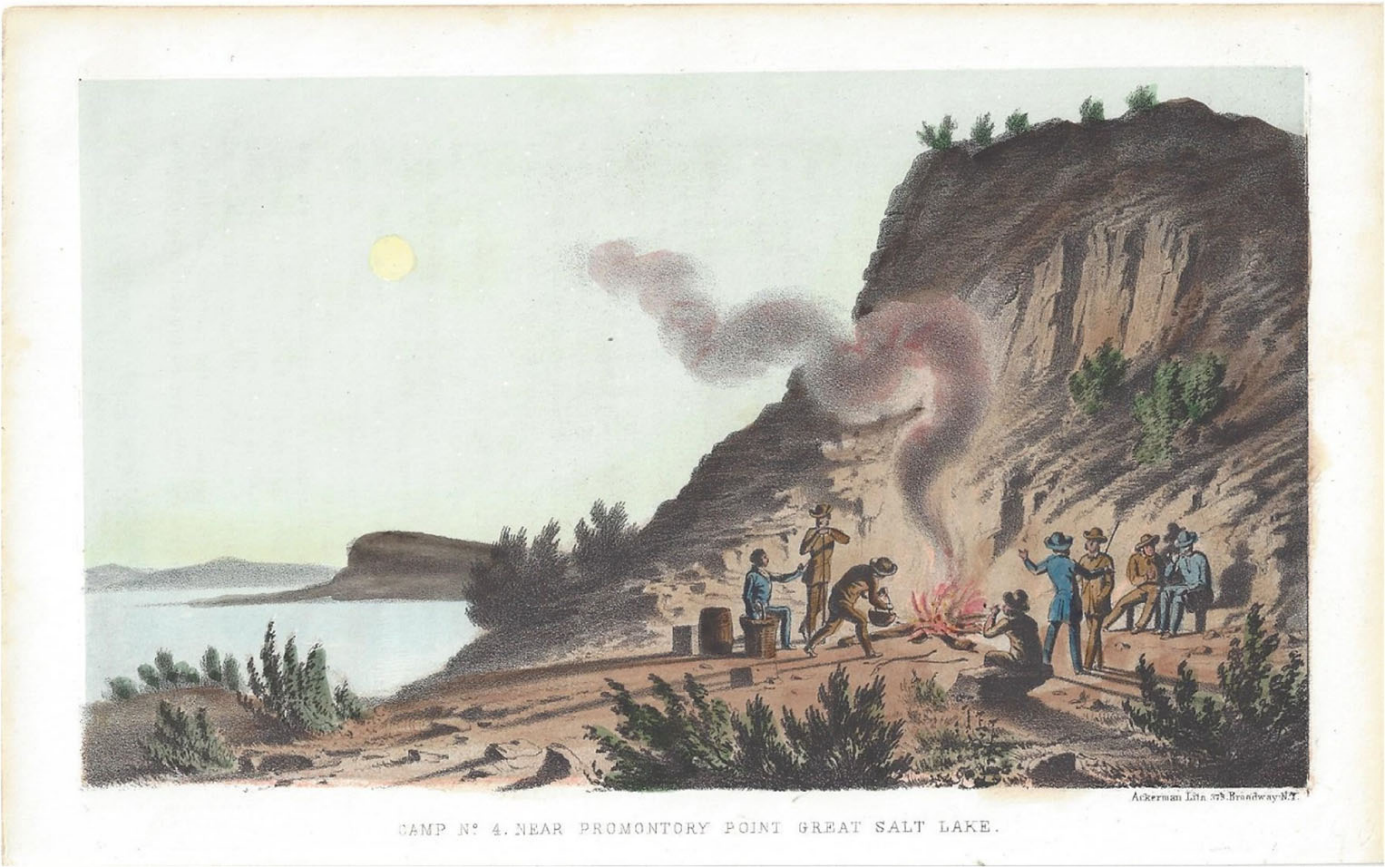
Stansbury company camp near Promontory Point. A lithograph from Captain Stansbury’s report shows camping along the shoreline of Great Salt Lake. Their exploration mapped the lake but was also the first extensive review of the biology, chemistry and geology (Stansbury 1855) (Image is public domain)

In 1849, Howard Stansbury, a civil engineer and a Captain for the US Corps of Topographical Engineers, brought a company to circumnavigate GSL and map its margins and islands. Over the next 3 years, these men completed a thorough work describing the lake’s geography, natural history, minerals, and water chemistry (Fig. 4) (Stansbury 1855). Stansbury’s company produced remarkably accurate maps and the first drawings of GSL area flora, fauna, and geology.

### Anthropomorphic impacts on GSL

As the Salt Lake City settlement grew, the lake was seen as a resource for industry. Mineral extraction, agriculture diversions, and short cuts for the railroad would eventually impact the pristine lake that the Goshutes revered, and Frémont and Stansbury so carefully described. These activities also created diverse habitats for microorganisms.

The GSL mineral extraction industry produces sodium chloride (road and softener salt), magnesium chloride (for steel production), and potassium sulfate (fertilizer), also diverts and dams water (Behrens 1980; Bingham 1980; State of Utah 2018a). The evaporation ponds of these industries, in the north arm and along the edges of the lake, create microbial habits along a salinity gradient as the minerals are concentrated. As one flies over these GSL salt company plants, you see the coloration of the different microbial communities in different ponds (Fig. 1c).

The construction of a rock-filled railroad causeway from 1955 to 1959 bisected GSL and isolated the north arm of the lake, restricting exchange and creating an artificial salinity gradient (Fig. 1a, b) (Baxter et al. 2005; Cannon and Cannon 2002; Madison 1970). The difference in salinity can create sub-habitats for different communities of microorganisms. The saturated hypersaline north arm is a rosy pink due to the carotenoid pigment-containing microorganisms that live there (Almeida-Dalmet et al. 2015; Baxter et al. 2005; Jones and Baxter 2017) (Fig. 1). While the south arm, which receives the freshwater input from the watershed, is less saline, currently around 15% (Greer 1971; United States Geologic Survey 2018). The diversity of algae is greater in the south arm [e.g. D'Adamo et al. 2014; Lindsay et al. 2017; Meuser et al. 2013], leading to a green and blue coloration (Fig. 1).

Other damming events created critical bird habitats and are part of the Western Hemispheric Shorebird Reserve Network, but they also diverted water from GSL and created a series of interesting microbial environments at a single site. As early as the late 1800s, damming and diversion of water upstream of GSL to create agricultural lands, resulted in other microniches around the lake such as freshwater marshes or brackish pools with a lower salinity. In 1928, the U.S. Congress passed an act to make the Bear River delta a National Wildlife Refuge (United States Division of Fish and Wildlife 2018). Later, federal agencies diked and dammed the Bear River, which flowed into the GSL north arm, to produce bird habitat there (United States Bureau of Reclamation 1962). The structures are on the margin of the lake and have been maintained over time, preventing inflow of this water to GSL. For similar reasons, the Farmington Bay Wildlife Management Area was constructed beginning in 1935, which created a brackish bay to the east of Antelope Island in GSL (State of Utah 2018b). Due to the lower salinity, relative to the rest of the lake, this area possesses a unique microbiology, and is prone to cyanobacterial blooms which are potentially toxic (Marcarelli et al. 2006).

## Microbiology of Great Salt Lake

### Historical observations and contradictions

The unique landscape, changing colors, fragrance, and salt crust beaches of GSL are certainly striking to the observer, and it is not surprising that the solitude of the place would conjure notions of stillness and lifelessness. Stansbury wrote, “Save the dashing of the waves against the shore absolutely nothing is heard. Not the jumping of a fish, the chirp of an insect, nor any of the least thing betokening life, unless it be that very rarely a solitary gull is disturbed in his midnight rumination and flys screaming away. All is stillness and solitude profound” (Fig. 4) (Stansbury 1855). The Captain was so convinced that the water was sterile and antiseptic (without microorganisms), he had his men experiment with using the GSL brine for salting meat for storage on the return trip home. The salt did indeed prevent spoilage, but gave a bad taste according to the men, and they referred to it as “salt junk.” The meat likely grew harmless microorganisms from the salty brine, as is the case with salted fish from which our first laboratory investigations of halophilic microbiology emerged (Browne 1922; Larsen 1986). This, along with the salt, would have augmented the flavor, hence the revulsion from the sailors.

Underscoring the idea of lifeless waters, Mormon pioneers continued to make parallels between GSL and the Dead Sea, an irresistible confirmation of Utah as a spiritual place (The Church of Jesus Christ of Latter-Day Saints 1997). Parley Pratt, exploring the valley for farming possibilities, just 2 days before Brigham Young’s wagon arrived, wrote in his journal that as they came closer to GSL, the soil had “a more sterile appearance.” Alfred Lambourne, an artist and writer who lived for a while on Gunnison Island in the middle of GSL, wrote: “At twilight a wild and thrilling spectacle….Dim and pale, the moon, the ghost of a dead world, lifted above the distant Wasatch peaks and stared at the acrid waters of a dead sea” (Lambourne 1909).

Though the water of GSL may at times appear dead, it is teaming with microbial life (Baxter et al. 2005) and actually, microorganisms enrich the waters of the Dead Sea as well (Oren 1999). Early human observers noticed smells and colors, but only later did they attribute this to the microbial communities. Frémont and his team described a *“disagreeable smell in stirring up the mud”* as they portaged a boat into the salty waters (Frémont 1845). Later, in 1877, John Muir had a more enjoyable take on the smell while swimming in GSL “…the cool, fragrant brine searches every fibre of your body…” (Muir 1877).

There are historical references to the color palette of GSL as well as the aroma. Frémont commented on the color, referencing the algae without realizing it, “The water continued to deepen as we advanced; the lake becoming almost transparently clear, an extremely beautiful bright green color…” (Frémont 1845). In fact, over time, many wrote about the seasonal changing colors of GSL, including Dale Morgan, “Visitors have called its waters bright emerald, grayish green and leaden gray; they have called them sapphire and turquoise and cobalt -- and they have all been right. Its color varies with the time of day, the state of the weather, the season of the year, the vantage point from which it is seen” (Morgan 1947). And we know now that the colors and smells do change over time, as the microbial communities shift in response to oscillations in temperature, lake level, and salinity gradients (Almeida-Dalmet et al. 2015; Baxter et al. 2005; Lindsay et al. 2017; Meuser et al. 2013; Rushforth and Felix 1982).

### Historical microbiology studies of Great Salt Lake

The nineteenth century brought pioneers, fortune seekers, and religious people to the Salt Lake valley, but it also brought scientists. Microbiologists were curious about what could survive in the brine of GSL, and there were attempts to isolate and describe life here through the mid twentieth century, when much of this investigation ceases to appear in the literature. Some of the early work was archived in bulletins, collections, and theses, and much of it was lost to later microbiologists relying on published literature.

**Fig. 5:**
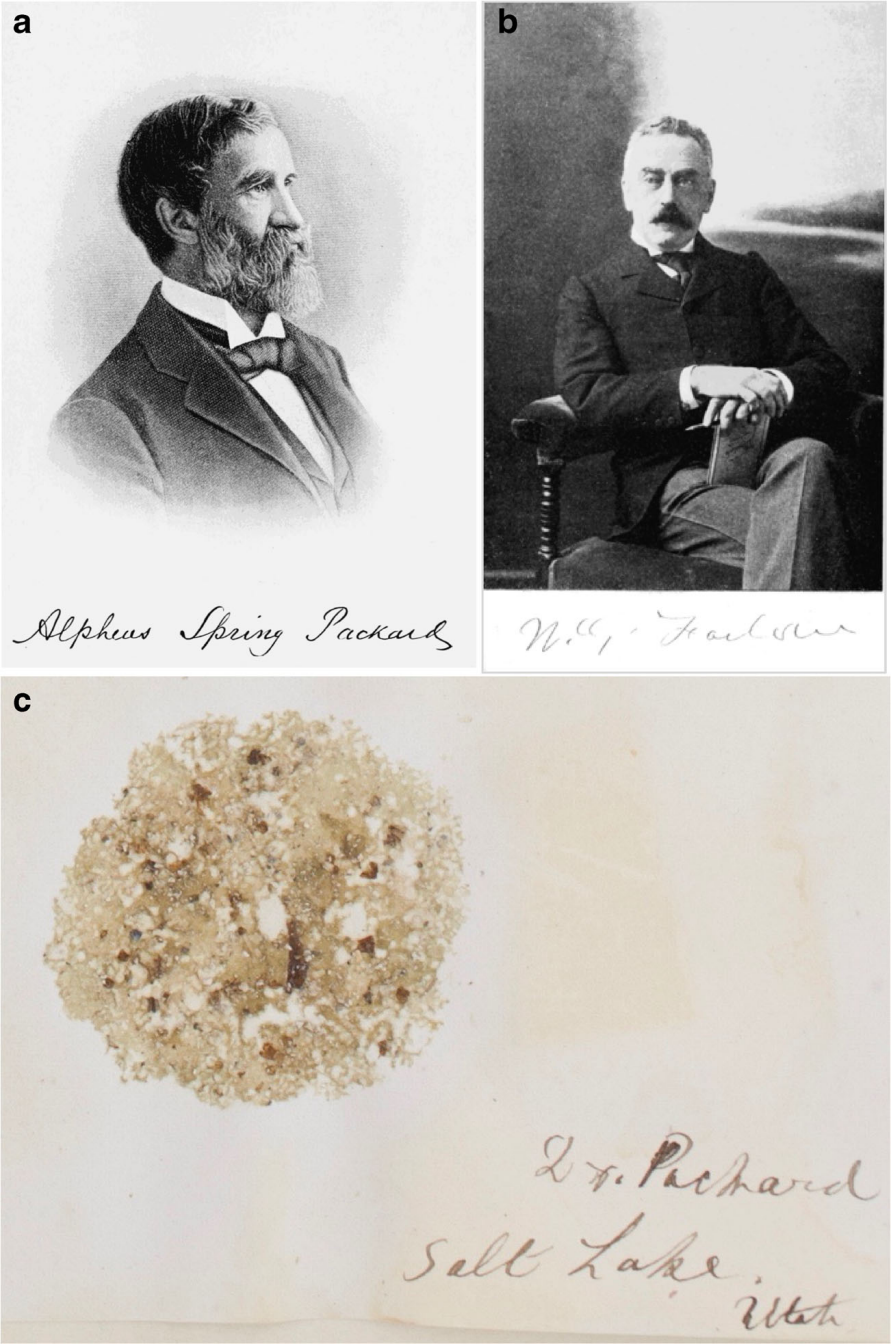
The first true microbiological exploration of Great Salt Lake. **a** Alpheus Spring Packard, who first sampled algae from the lake (Cockerell 1920) (Image is public domain). **b** William Gilson Farlow, who is reportedly the first scientist to isolate and identify microorganisms from Great Salt Lake samples sent to him by Packard in 1871 (Spencer 1904) (Image is public domain). **c** Dried sample of *Polycystis packardii* Farlow; accompanying notes (Harvard University 2018) indicate Packard collected samples as part of the Hayden expedition (Hayden 1873) (Image reprinted by permission of the Farlow Herbarium, Harvard University, Cambridge, Massachusetts)

The first microbial isolations from GSL trace back to Alpheus Spring Packard Jr. (Fig. 5a), who was a member of the Ferdinand Vandeveer Hayden expedition, a geological and biological study of the western US territories in the 1870s (Hayden 1873). Packard, who later became a professor of zoology and geology at Brown University (Rhode Island), was an expert in moths who had more than 100 species attributed to him and was mainly a part of the team due to his entomological expertise (Cockerell 1920). While in Utah traveling by stagecoach, he collected algae samples and in 1871, sent them to botanist, William Gilson Farlow (Fig. 5b), to characterize (Spencer 1904). Later described in a journal, “Professor W.G. Farlow, of Harvard University, soaked out and examined the dried material, which he found to consist largely of grains of sand and remains of small animals, mixed with which were three species of Algae” (Crisp et al. 1880). Farlow isolated and identified these three species of algae in his laboratory: the cyanobacteria *Polycystis packardii, Rhizoclonium* sp., and *Ulva marginata* (Crisp et al. 1880; Harvard University 2018; Packard Jr. 1879). The latter two species likely occur only in freshwater seeps and marshes (Rushforth and Felix 1982). These algae samples were pressed and are currently stored in the Farlow Herbarium (Fig. 5c) (Harvard University 2018).

A little later, a geologist, Rothpletz, connected cyanobacteria (*Gleothece* and *Gleocystis*) to the carbonates in the lake (Rothpletz 1892), pointing to the role of the microbiota in carbonate formation. At the time, his work was suspect: “We have been unable to find [these species] in the part of the lake studied, and it might be said, too, that the connection between these and the ooliths has not been generally accepted, even by geologists” (Dainels 1917). We now have a deeper understanding of the role of cyanobacteria in carbonate formation [e.g., (Lindsay et al. 2017)], which may validate Rothpletz’s hypothesis.

**Fig. 6:**
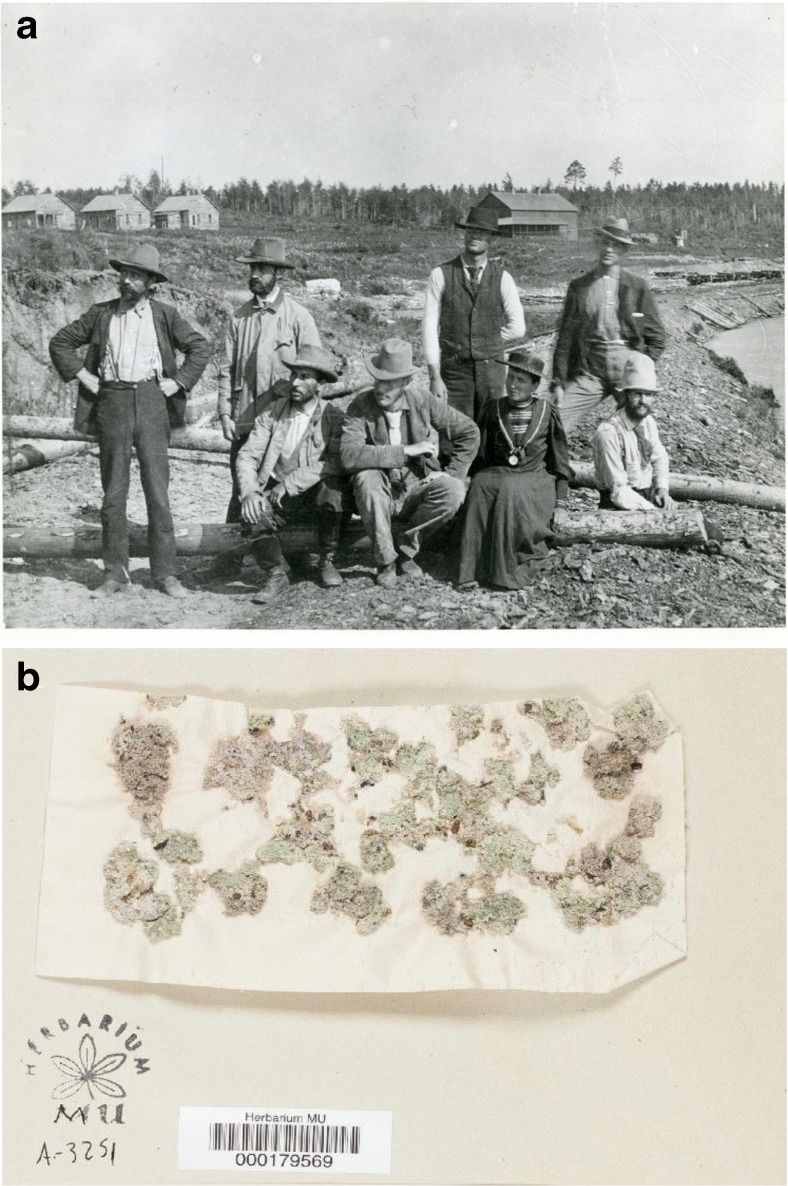
Josephine Tilden, one of the first researchers to study Great Salt Lake microbiology. **a** Tilden, donned in a dress, working with other scientists in 1893 (image reprinted by permission of University of Minnesota). **b** Tilden’s preserved specimen of *Aphanothece utahensis* Tilden (Macroalgal Herbarium Portal 2018; Tilden 1898) (image reprinted by permission of the W.S. Turrell Herbarium, Miami University, Oxford, Ohio)

Perhaps the very first on-site systematic study of microorganisms in GSL was in the 1890s by a remarkable woman, Josephine Tilden, the first female professor at University of Minnesota and an algae specialist (Horsfield 2016). She embarked on a science exploration of the west, sampling and isolating algae from extreme environments, including Yellowstone and Great Salt Lake (Fig. 6a) (Tilden 1898; Tilden 1910). Tilden reported five species of algae from the lake: *Aphanothece utahensis* Tilden*, Polycystis packardii* Farlow*, Dichothrix utahensis* Tilden*, Enteromorpha tuhulosa* (Kiitzing) Reinbold*, and Chara contraria* Braun. In addition, she stored samples in herbariums which are still available today (Fig. 6b) (Macroalgal Herbarium Portal 2018). In her description of the sampling site (Garfield Beach) for *Polycystis packardii* Farlow, Tilden included details that provide significant context, “Forming irregularly-shaped balls of masses of a firm gelatinous structure, showing various tints of pink, brown and green. In thick masses around edge of lake for a distance of forty feet out from shore and one to two feet in depth. Often washed ashore and left in beds on sand” (Tilden 1898). Several scientists who followed subsequently isolated Tilden’s *Aphanothece* species from GSL brine [e.g., Flowers 1934; Kirkpatrick 1934].

In the same decade as Tilden’s work, looking to farm oysters in GSL, biologist Henry Moore authored a study that also commented on the presence of diatoms at freshwater interfaces, which would work to feed the shellfish (Moore 1899). A Mormon religious leader and geologist, James Talmage, wrote about diatom colonies along the shore and in the water column (Talmage 1889; Talmage 1900). Lyman Daines, a graduate student and then professor at the University of Utah was able to culture GSL diatoms in the laboratory in 1910 (Daines 1910; Daniels 1917). All of these studies pointed at the question of whether the discovered diatoms washed in from freshwater or if they were truly hypersaline. The most complete early study of diatoms was done by Ruth Patrick, who surveyed these algae around the lake and in cored sediment samples, noting many species that were vestige paleo-remains, some that were freshwater in areas with springs or river input, and some that were hypersaline (Patrick 1936). As a young woman in science, Patrick at the time of this study was working as an unpaid “volunteer” for the Academy of Natural Sciences (ANS) (Academy of Natural Sciences 2018). Over the next decade, she became a paid employee and one of the world’s diatom experts, and the ANS limnology research center was named in her honor in the 1980s. Recent studies confirm the presence of Patrick’s hypersaline diatoms (Lindsay et al. 2017).

Green algae such as *Dunaliella viridis*, which are important for the survival of *Artemia* (Belovsky et al. 2011; Rushforth and Felix 1982) were observed in 1910 by Daines but called *Chlamydomonas* sp. at that time (Daines 1910; Daniels 1917), “The presence of plants [sic algae] is not so evident to the casual observer, although, at certain times of the year, clumps of greenish material, which must at least suggest a vegetable growth, are very plentiful.” Others isolated and described these chlorophytes in detail later on [e.g., Kirkpatrick 1934], and we now know their significance and enormous abundance in the overall GSL ecosystem (Belovsky et al. 2011). The brine shrimp thrive when the glycerol-packed *Dunaliella* is available (Fig. 3). Later we learned that at salt saturation, a carotenoid-containing orange species, *Dunaliella salina*, inhabits the hypersaline north arm (May 1978).

**Fig. 7:**
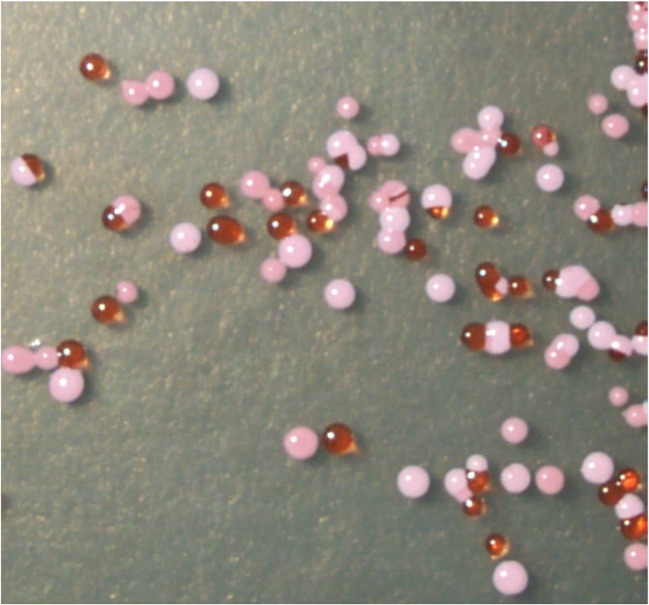
Great Salt Lake halophilic archaea colonies growing on solid media. Brine from the north arm of Great Salt Lake was recently inoculated on high salt agar media. Carotenoid-containing microorganisms appeared after about 2 weeks. Early researchers noted the beautiful colors of their isolates (Daines 1910; Frederick 1924; Kirkpatrick 1934; Smith 1936), and later researchers described the roles of the pigments in photoprotection (Jones and Baxter 2017) (Image credit: B.K. Baxter)

The first reference to prokaryotic life (outside of cyanobacteria) was from Daines in his thesis (Daines 1910): “The fact that putrefaction and decay are taking place in the water, especially near to shore, where organic material is abundant, shows conclusively that bacteria are present.” He performed colony counts from plating brine, from a south shore site, on salt agar and determined that the concentration was between 200 and 625 culturable cells per milliliter. Daines also isolated five species of bacteria/archaea, three of which were producing “abundant pigment” colored yellow, orange and violet. These carotenoid pigments are by now well-associated with photoprotection in halophilic archaea (Fig. 7) (Jones and Baxter 2017), but this may be the first notation of such. In 1924, Elfriede Frederick published a thesis describing bacteria from GSL (Frederick 1924). From brine samples, she cultivated and identified 12 strains, including the pink-colored “*Serratia salinaria*,” a halophilic archaea species found world-wide in salty places, and later renamed *Halobacterium salinarum*. The coloration of regions of GSL by halophilic archaea as seen in Fig. 1 (a and b) was rarely remarked upon by the scientists working on the lake before the causeway was installed. Tilden described specific areas of the lakeshore colored red (Tilden 1898), and others commented on the pigmented isolates from GSL (Daines 1910; Frederick 1924; Kirkpatrick 1934; Smith 1936), but pink water is not discussed. The periodic high salinity of the lake was likely not a stable enough condition in which the halophilic archaea could thrive, until the separation of the north arm which allowed the water to be at salt-saturation year-round.

**Fig. 8:**
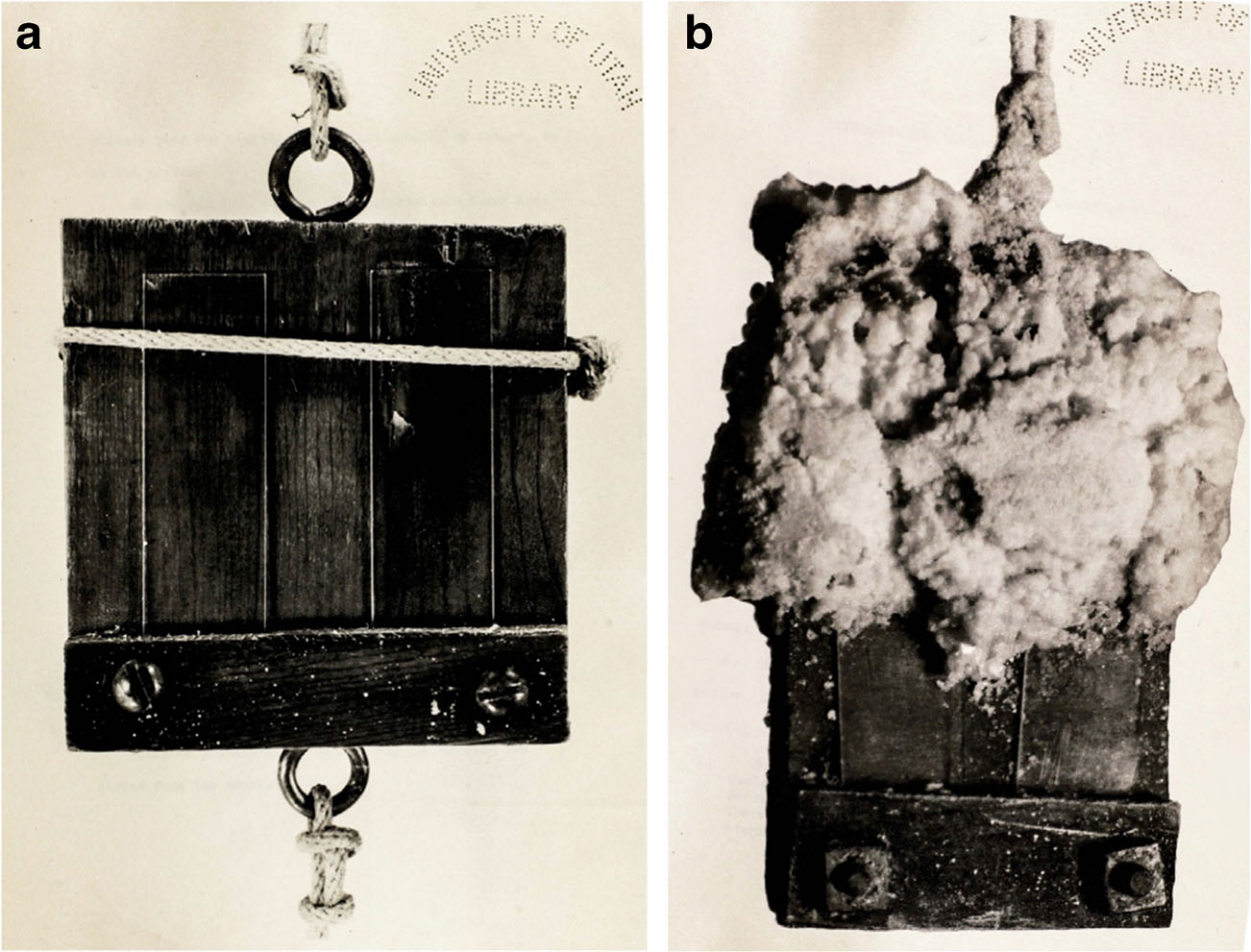
Slide sampling apparatus. **a** The apparatus used for incubating slides in marine water fashioned by Claude Zobell and photographed for Winslow Smith’s thesis (Smith, 1936). **b** A replica of the Zobell apparatus built by Smith after he removed it from the period of soaking in Great Salt Lake during a time of high salinity. (Images reprinted by permission from Special Collections, J. Willard Marriott Library, University of Utah)

Daines became a professor of bacteriology at the University of Utah, and was listed as a committee member for Winslow Smith, whose 1936 master’s thesis was an exploration of the idea of sterility in GSL (Smith 1936). He surveyed the halophilic microbiology literature of the time and hypothesized that GSL would have prokaryotic life in all areas, despite localized high salinity conditions. He began a collaboration with Claude Zobell, a marine microbiologist at the Scripps Institution of Oceanography in La Jolla, California. Zobell used a technique of submerging slides in water onsite for a period then staining and counting colonies that grew on the slides (Fig. 8a) (Zobell et al. 1937) This technique, for the first time, gave visual information about communities rather than isolated species. Smith argues for studying communities: “…pure culture studies and the usual culture methods present only fragmentary information about what certain bacteria do when freed of competition for life and placed in an ideal but unnatural environment” (Smith 1936). Smith manufactured a similar device and was astounded by the salt crystals that permeated the slide holder when he reeled in the apparatus (Fig. 8b). He reported that not a single sampling location resulted in sterile samples. It seemed as if GSL was teaming with microorganisms (Smith 1936; Zobell et al. 1937). However, Smith missed one important observation when he threw away plates that “were overgrown with mold” without realizing he may have disposed of the first evidence of fungi in GSL. We now know that fungi are part of the lake’s microbial community (Baxter and Zalar 2018).

Smith (Smith 1936) also observed the same colorful archaea described by Daines (Daines 1910) and Kirkpatrick (Kirkpatrick 1934), “The colonies that developed on these different media were highly bizarre. Violet, red, orange, tan, brown and yellow colonies were to be observed as well as white and colorless ones. Many of them were of picturesque morphology that defies description.” Smith, working with Zobell, focused on recording some environmental parameters for the first time, such as: dissolved oxygen, osmotic pressure, ion concentrations, wave action, and freshwater inputs. He went to great lengths to do careful sterility controls on all sampling and culturing materials such that he could confirm the presence of microorganisms in his work was indeed from GSL. Smith also was concerned with the sewage that was being dumped into GSL, and he designed experiments to test for the viability of pathogens in lake water, concerned with the fact that humans were recreating and soaking in the lake’s many resorts. Working with Zobell, Smith determined that enteric bacteria, like *Escherichia coli*, were killed by the hypersaline water (Zobell et al. 1937). Later this issue was contested with new membrane filter technique, and once again concerns were raised about raw sewage entering the lake as *E.coli* was shown to persist with this method (Fraser and Argall 1954).

The first reference to protozoa, in 1917, was by a zoologist at the University of Utah, Charles T. Vorhies, who was interested in the diets of brine shrimp and brine flies (Vorhies 1917). He described several protozoan species including an amoeba (likely *Amoeba flowersi* Jones), a ciliate (likely a *Uroleptus* species), and a species of Euglena (likely *Euglena chamberlini* Jones). Shortly after, Dean A. Pack, a Botany instructor, described *Uroleptus packii* and *Prorodon utahensis* (Pack 1919). Using dilution methods, Pack noted changes in the protozoa as he lowered salinity. The protozoa were re-discovered and better described by D.T. Jones in 1944 (Jones 1944), who named the Euglena for Ralph Chamberlin, a prolific biologist at the University of Utah and one of his mentors. Chamberlin was a taxonomist known not only for naming thousands of invertebrates, but also for building the University’s zoology department and medical school (Wintrobe 1982). Jones named the Amoeba he was studying after Seville Flowers, a colleague and botanist who had first found the salty species (Behle 1984; Jones 1944). Frederick Evans and collaborators later worked on the protozoa and found several new yet unidentified species and described eight south arm protozoan taxa (Evans 1958; Evans 1960; Evans and Thompson 1964; Flowers and Evans 1966), and Reddy described a GSL ciliate, *Euplotes*, in a later thesis (Reddy 1971). The only reference to north arm hypersaline protozoa was from Post in 1977 (Post 1977), who observed them in aquaria microcosms, but these were not his primary area of study and were not identified. Sadly, the field of protozoa of GSL has not progressed since this time.

**Fig. 9:**
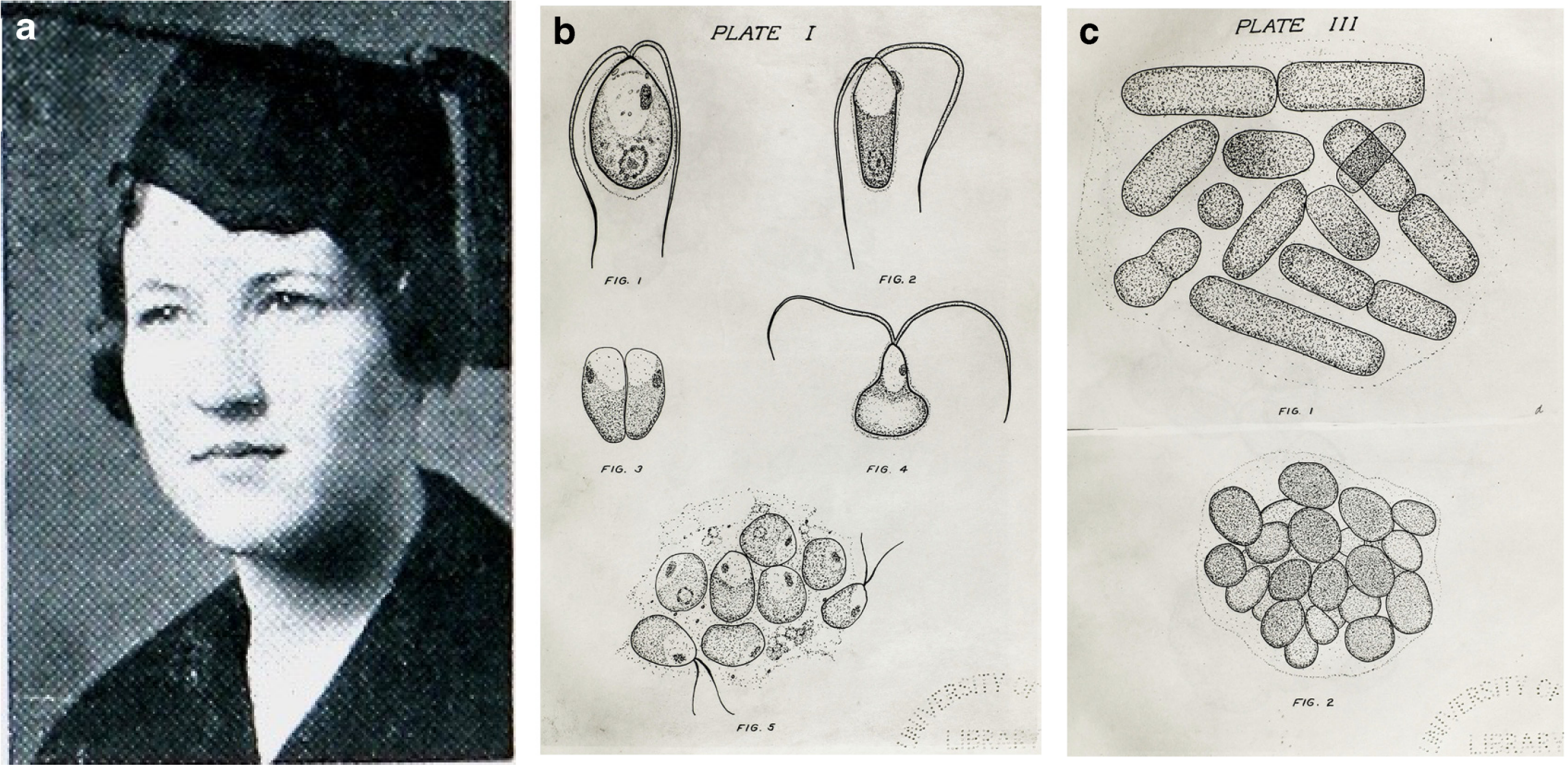
Ruth Kirkpatrick and her illustrations (Kirkpatrick 1934). **a** Kirkpatrick’s 1933 yearbook photo in which she is listed as a member of the Pi Beta Phi women’s academic fraternity and as “Engineer Queen.” **b** Plate I from Kirkpatrick’s thesis depicts her original drawings of various forms of *Dunaliella viridis* (referred to as “*Chlamydomonas*” at the time) magnified 400 times. **c** Plate III from the thesis contains carefully stippled images of *Aphanothece* forms “c” and “d,” magnified 1000 times. Kirkpatrick stated of her sampling site for this cyanobacterium, “In October, the covering was so dense that the whole rocky shoreline on the east side of Antelope Island appeared red.” (Images reprinted by permission from Special Collections, J. Willard Marriott Library, University of Utah)

In addition to his work on invertebrates, Ralph Chamberlin also mentored some GSL microbiology graduate student projects [e.g., Kirkpatrick 1934; Smith 1936]. In the 1930s, Chamberlin served as a mentor for Ruth Kirkpatrick (Fig. 9), who earned a Masters in the biology department at the University of Utah (Kirkpatrick 1934). Previously, as an undergraduate engineering major, in 1931 she was crowned “Queen of the Engineers” on the same night that her male colleagues were elected officers (The Salt Lake Tribune 1931). Her subsequent master’s thesis reviewed the GSL microbiology literature well and added some important in-depth observations (Kirkpatrick 1934). To the notion regarding freshwater microorganisms that may have been incidental in GSL sampling due to stream flows into the lake, “…the writer believes the only possible way to determine the species normally present in Great Salt Lake is by growing the algae in cultures over a period of time, long enough to eliminate any forms that may have come from extraneous sources.” Thus, this was her approach. Kirkpatrick gave credit to Daines (Daines 1910; Daniels 1917) for using good cultivation practices, and she used these techniques to observe cultures over prolonged periods under conditions varying salinity (Kirkpatrick 1934). She also was the first to collect microorganisms both spatially and temporally. She noted changing salinity of GSL over seasons and at different sites affecting what researchers could isolate at different times in various studies. In general, Kirkpatrick’s work was done at a time when the lake level was low, and the salinity measured as much as 26%, which was very high since this was before the construction of the causeway that created the isolated north arm. She collected from sediment and water at Black Rock beach on the southern shore of GSL, at the east shore near Syracuse, and off the west shore of Promontory Point. She also sampled exposed “algae covered rocks” (likely microbialites) at Antelope Island. She worked out culturing conditions for diatoms (*Navicula*), green algae (*Dunaliella*) (Fig. 9b), and various protozoa. Kirkpatrick described five “colonial forms” of cyanobacteria (Fig. 9c) linking these back to prior studies and suggesting that *Aphanothece utahensis* and *Polycystis* (*Microcystis*) *packardii* co-colonize in some areas, making their distinction difficult. She noted that most of the isolated strains did not thrive in the laboratory, an important observation from microbiologists at that time, which led future scientists studying microbial diversity to adopt non-cultivation methods of study decades later [e.g., Almeida-Dalmet et al. 2015; Lindsay et al. 2017; Meuser et al. 2013].

In the middle of the twentieth century, the center of studies of microbiology of GSL, the University of Utah, ceased to sponsor GSL projects with such frequency. Likely, key faculty retired. Also, the department of bacteriology collapsed into the biology department at some point, and in the early 1970s, a clinical microbiology department, focused on pathology, appeared at the university’s medical school (University of Utah 2018). No longer were aquatic biologists exploring GSL in Utah in a time when microbiological methodology was improving. In 1959–1961, the Union Pacific railroad causeway was built to bisect GSL (Cannon and Cannon 2002; Madison 1970). As discussed above, this separation changed the lake’s salinity gradient and thus, its microbiology. Studies after this time must contain the caveat of this change in the ecosystem.

Post-causeway researchers often separated their studies spatially with respect to the north arm, which immediately began its approach towards perpetual salt-saturation, and the south arm, for which salinity was decreased, resulting in an observed increase in microbial diversity (Evans and Thompson 1964; Rushforth and Felix 1982). There also was a concern that organic matter was accumulating in the hypersaline north arm due to a lack of bacterial degraders (Post 1977). The state of Utah welcomed the brine shrimp industry to the south arm as it ramped up in the 1970s (Wotipka 2014) and brought a new economic interest in the lake’s health. Doyle Stephens worked as a research hydrologist for the US Geological Survey, and he was a GSL researcher who approached the lake from several different angles. He had an undergraduate degree in biology, a master’s in entomology and his PhD in limnology (FRIENDS of Great Salt Lake 2018). He spent his career focusing on the sustainable management of the GSL ecosystem, including the economically important brine shrimp industry and the algae that feed these tiny crustaceans. Stephens, as an expert on the unique ecology of terminal lakes, was the right scientist to monitor the lake’s changing salinity with the construction of the railroad causeway (Stephens 1974; Stephens 1990; Stephens 1998; Stephens and Gillespie 1972; Stephens and Gillespie 1976). His work with the brine shrimp industry (Great Salt Lake Artemia Association 2018) for the Utah Division of Wildlife’s Great Salt Lake Ecosystem Program (State of Utah 2018c) set up a collaborative science-based management strategy that still exists today (Belovsky et al. 2011). This approach, focused on measuring the abundance of brine shrimp and their cysts as well as the distribution and types of phytoplankton, resulted in an ongoing, predictive model for the state which regulates the industry.

During this time of a changing lake and a growing industry, there were two important contributors to the microbiological literature on GSL. Sam Rushforth, a biology professor at Brigham Young University and then Utah Valley University, led many studies describing, in particular, the algae of the lake in the changing context of salinity (Felix and Rushforth 1977; Felix and Rushforth 1979; Felix and Rushforth 1980; Rushforth and Felix 1982; Rushforth and Merkley 1988). He and colleagues carefully traced the historical contributions (Rushforth and Felix 1982) and cataloged the algae discovered at that time (Rushforth and Merkley 1988). Further north at Utah State University in Logan, Fred Post was a microbiologist who entered GSL studies because there was so little work being done in the 1970s. He characterized several isolates of halophilic archaea and bacteria and published a few articles including important reviews (Cronin and Post 1977; Post and Stube 1988; Post 1975; Post 1977; Post 1980a; Post 1980b; Post 1981; Post et al. 1983). He investigated ecological phenomena such as gas domes in the salt crust (Post 1980b) and nitrogen utilization (Post and Stube 1988). Post remained focused on prokaryotes, but he recorded and published other observations that noted viruses (Post 1981), protozoa (Post et al. 1983), and a fungal species in brine-soaked wood (Cronin and Post 1977). With his location in the northern part of the state, he focused on the north arm more than any other researcher had up until that point in time (Post 1980a, 1981), in part because the hypersalinity there since the construction of the causeway was still a relatively new condition. Post retired before molecular techniques like gene and genome sequencing were common, and before the Domain of archaea was understood and readily applied to taxonomy (Woese and Fox 1977), but he laid the groundwork for studying halophilic archaea from GSL.

### Recent microbiology studies of Great Salt Lake

**Fig. 10:**
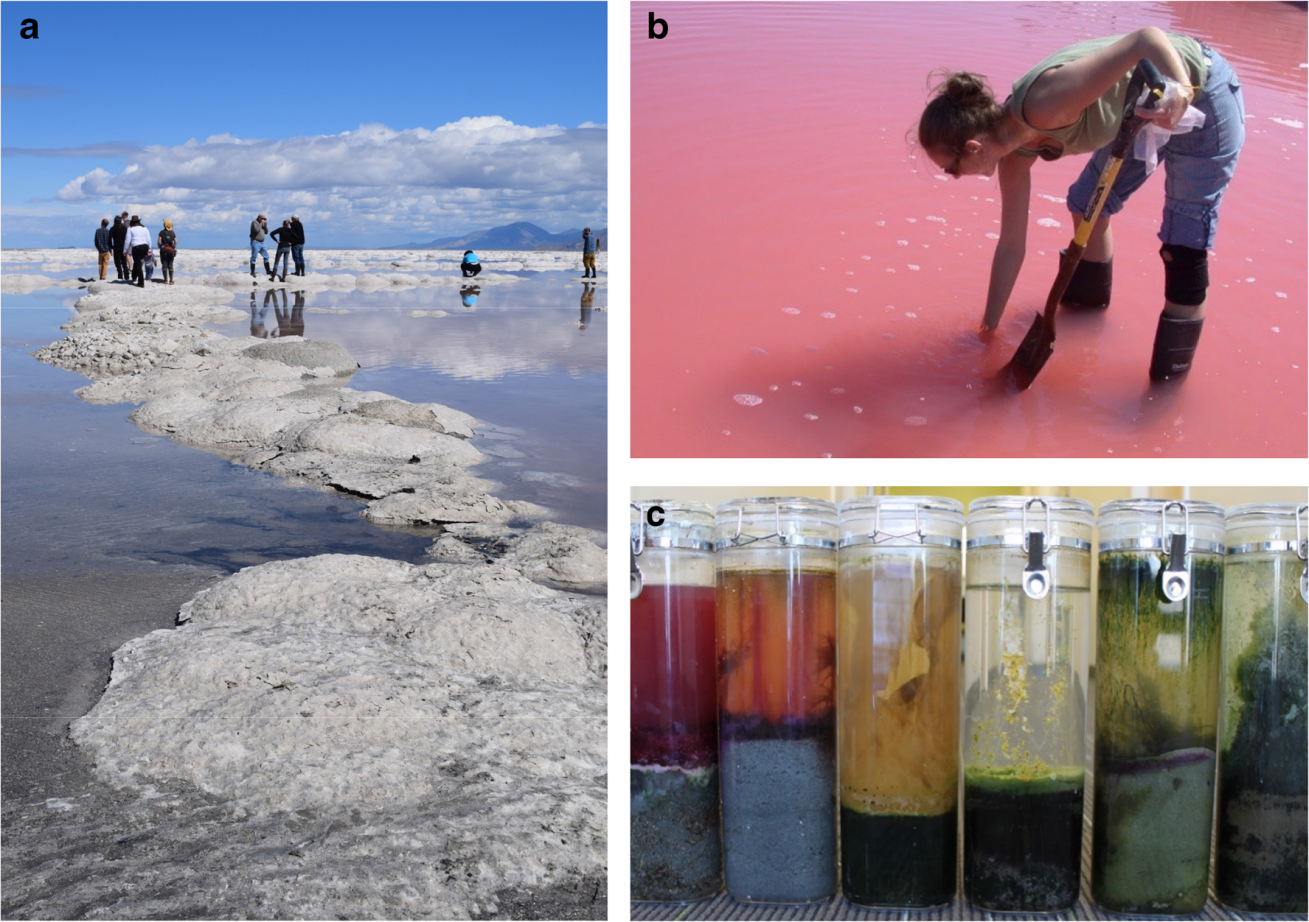
Current research on Great Salt Lake is interdisciplinary and focused on microbial communities. **a** Geologists, chemists and biologists work together in the field to study the salt-encrusted microbialite vestiges in the north arm. **b** Great Salt Lake Institute student harvests halite crystals for astrobiology studies from the richly colored water of a salt evaporation pond. **c** Microbial communities, sampled from a variety of locations around Great Salt Lake, form biofilms and mats in vitro as they grown in Winogradsky columns (Image credits: Great Salt Lake Institute)

The historical efforts to build an understanding of GSL microbial diversity seemed to lose momentum during the molecular revolution in the 1980s that would color modern studies. This author began working in Utah in 1998, and recent microbiology studies on GSL were scant or non-existent. I met with Fred Post, then retired, with my notion of collecting GSL halophilic archaea to use as DNA repair models (Baxter et al. 2007; Jones and Baxter 2016, 2017). Post sent me to the lake to find my model microorganisms as his were destroyed, after he retired, in a freezer-cleaning incident, which he reported with much sadness. With Post’s maps in hand, I began working on GSL microbiology with molecular training but little to no skill in microbial ecology. The dearth of work brought other Utah scientists to the table from Brigham Young University (Shen et al. 2012; Tazi et al. 2014), Weber State University (Shen et al. 2012), and Utah State University (Parnell et al. 2009, 2010, 2011; Weimer et al. 2009). We were building momentum, but it was clear that there was much work to do to understand the microbial foundation of this iconic ecosystem. Intentionally, I reached out to other researchers around the world who studied halophilic microorganisms elsewhere (Baxter et al. 2005; Baxter and Tate-Wright 2018). Ten years ago, we started Great Salt Lake Institute at Westminster College (Westminster College 2018), which carried the mission of facilitating large research projects on the lake, including helping scientists with access to sampling sites and logistical aid (Fig. 10). As a result, we have engaged researchers in our state with scientists bringing new ideas, insights and techniques to GSL. Whether facilitated by our institute or driven independently by current scientific questions, GSL microbiology is now an active area of research. A current review of the known GSL genera of bacteria, archaea, and eukaryotic microbial life is in press (Baxter and Zalar 2018).

Cultivation studies of GSL microorganisms are still relevant, as one can isolate lab strains to explore physiology and biochemistry (Almeida-Dalmet et al. 2018; Baxter et al. 2007; Baxter and Zalar 2018; D'Adamo et al. 2014; Pugin et al. 2012). Researchers have cultivated a number of GSL strains of bacteria and archaea, isolating in laboratory conditions in salty media, to study their physiology and genetics. There are currently 14 GSL strains, one archaeal 13 bacterial, stored and maintained in the following culture banks: the American Type Culture Collection (ATCC, USA); the Biological Resource Center (NBRC, Japan), the Leibniz Institute DSMZ (DSM, Germany), and the All-Russian Collection of Microorganisms (VKM, Russia) [Reviewed in Baxter and Zalar 2018].

Current researchers have elucidated much about the microbial communities of the lake from molecular work. Studies that utilize techniques that assess the DNA of an environment, though the SSU rRNA genes or from metagenomes, give a more complete depiction of the community members since these methods detect the species that are not culturable. From such studies, we know that the microbial communities in GSL are composed predominantly of halophilic archaea and bacteria (Almeida-Dalmet et al. 2015; Baxter et al. 2005; Meuser et al. 2013; Parnell et al. 2011; Tazi et al. 2014; Weimer et al. 2009). We now understand that assemblages of microorganisms must be dynamic, responding to the changes in salinity and temperature that accompany the seasons GSL experiences (Almeida-Dalmet et al. 2018). Salinity gradients in the less saline south arm of the lake have been shown to influence the composition of planktonic species (Boyd et al. 2014; Lindsay et al. 2017; Meuser et al. 2013). However, the hypersaline north arm microbial communities are more stable over time and not as impacted by changes in temperature and salinity (Almeida-Dalmet et al. 2015). These stable groups of microorganisms also have a lower phylogenetic diversity relative to communities in the south arm (Parnell et al. 2009, 2010, 2011). Though we still do not know much about the specific roles of microorganisms in GSL, we are concerned about their role in the bioaccumulation and modification of heavy metal pollutants (Boyd et al. 2014; Wurtsbaugh et al. 2011), a particularly difficult problem to solve in a terminal lake.

Modern studies have broadened our knowledge of life forms in GSL and how they work together. We now understand that viruses are plentiful (Baxter et al. 2011; Motlagh et al. 2017; Shen et al. 2012), especially in the north arm where they are the only predators of the bacterial and archaeal residents (Baxter et al. 2011). GSL Fungi were ignored in the past, but recently 32 strains of fungi were isolated from both north and south arms (Baxter and Zalar 2018) though we do not know what role they play in the communities. We have learned that GSL halophilic archaea are resistant to desiccation and UV radiation as well as high salinity (Baxter et al. 2007; Jones and Baxter 2016; Jones and Baxter 2017), and GSL bacteria may tolerate high temperatures or high pH (Pugin et al. 2012), making GSL halophiles polyextremophiles. We have begun thinking about GSL microorganisms as members of richer assemblages or microorganisms that work together in a community (Fig. 10c) [e.g., Almeida-Dalmet et al. 2015; Lindsay et al. 2017; Meuser et al. 2013].

And we have collaborated across disciplines to understand how geology and biology work together in GSL (Fig. 10a). One example of this is the calcium carbonate microbialite structures that line the lake bottom, which are likely precipitated from the action of photosynthesis, and resulting localized chemistry changes, by their associated cyanobacteria (Chidsey Jr et al. 2015; Lindsay et al. 2017). These mounds grow and power the lake, converting solar energy and forming a critical component of GSL primary production. In addition, the formation of halite (salt) and gypsum crystals, in the hypersaline sediment (Fig. 10b), may preserve GSL life forms over time, and this provides a link to potential mineral biosignatures on Mars (Perl et al. 2016). Indeed, GSL as a Martian analogue brings these historical studies into the future.

## Insights

GSL had a human history, from more than 13,000 years ago until almost 200 years ago, that was not recorded in writing, a caveat of historical studies. We have limited information about the native people of the GSL area and their relationship to the lake. I remain curious about their observations about colors and smells that would have revealed GSL microbiology. Author and advocate for American Indians in Utah (Cuch 2000), Forest Cuch reported “I was told by a Ute elder, that the Utes complained about the brine odor from Great Salt Lake. Therefore, they usually camped far from the lake closer to the Wasatch Mountains when they were in the area.” (Personal Communication to the author). I would like to underscore that the history of GSL did not begin with the entrance of white people and their process of science. There were already people in the valley who likely understood the vacillations, smells, and color changes of their terminal lake as it swelled and shrank over the years. As European-Americans moved into the western US, their manner of exploration and science followed. Much of what we know is based on such written records because that is what we have available, and this becomes the focus of our science history.

The historical scientists in this field, who explored this “Dead Sea,” and found it very much alive, inspired future GSL scientists to apply their evolving methods. Their diligence in the lab, careful note-taking, patience, and preservation of samples support the work we do today on a much different ecosystem. Modern GSL is changing, in ways that reflect upstream water demands of a growing population and climate change. Our lake is shrinking, and there may come a time when we no longer see the oscillations of lake elevation but only a downward trajectory as have other saline lakes in the world (Wurtsbaugh et al. 2017). It is therefore critical that we continue to study the microbiology of GSL in a temporal fashion.

This author has been particularly touched by the stories of the pioneering women in the field, like Tilden, Patrick, Frederick and Kirkpatrick. Working at a time when few women were in science, they persevered and contributed, some without a salary nor much recognition. It is to these women that I dedicate this work.

## References

[CR1] Academy of Natural Sciences (2018) Dr. Ruth Patrick. In: The Academy of Natural Sciences. http://www.ansp.org/research/environmental-research/people/patrick/biography/. Accessed 7 March 2018

[CR2] Aldrich JM (1912). The biology of some western species of the dipterous genus *Ephydra*. J N Y Entomol Soc.

[CR3] Aldrich TW & Paul DS (2002) Avian ecology of Great Salt Lake. In: Gwynn, JW (ed) Great Salt Lake: An Overview of Change. Salt Lake City, pp 343–374

[CR4] Almeida-Dalmet S, Sikaroodi M, Gillevet PM, Litchfield CD, Baxter BK (2015). Temporal study of the microbial diversity of the North Arm of Great Salt Lake. Microorganisms.

[CR5] Almeida-Dalmet S, Litchfield CD, Gillevet P, Baxter BK (2018). Differential gene expression in response to salinity and temperature in a *Haloarcula* strain from Great Salt Lake. Genes.

[CR6] Atwood G, Wambeam TJ, Anderson NJ, Oviatt CG, Shroder JF (2016). The present as a key to the past: Paleoshoreline correlation insights from Great Salt Lake. Lake Bonneville a scientific update.

[CR7] Bagley W (2010). So rugged and so mountainous: blazing the trails to Oregon and California 1812–1848.

[CR8] Barnes BD, Wurtsbaugh WA (2015). The effects of salinity on plankton and benthic communities in the Great Salt Lake, Utah, USA: a microcosm experiment. Can J Fish Aquat Sci.

[CR9] Baxter BK and Tate-Wright K (2018) Carol D. Litchfield: Salt of the Earth. In: Whitaker RJ & Barton HA (eds) Women in Microbiology, American Society for Microbiology. 10.1128/9781555819545

[CR10] Baxter BK, Zalar P, Seckbach J, Rampelotto PH (2018). The extremophiles of Great Salt Lake: complex microbiology in a dynamic hypersaline ecosystem. Model ecosystems in extreme environments.

[CR11] Baxter BK, Litchfield CD, Sowers K, Griffith JD, Dassarma PA, Dassarma S, Gunde-Cimerman N, Oren A, Plemenitaš A (2005). Microbial diversity of Great Salt Lake. Adaptation to Life at High Salt Concentrations in Archaea, Bacteria, and Eukarya. Cellular Origin, Life in Extreme Habitats and Astrobiology.

[CR12] Baxter BK, Eddington B, Riddle MR, Webster TN, Avery BJ, Hoover RB, Levin GV, Rozanov AY, Davies PCW (2007). Great Salt Lake halophilic microorganisms as models for astrobiology: evidence for desiccation tolerance and ultraviolet radiation resistance. Instruments, methods, and missions for astrobiology X, 6694:669415.

[CR13] Baxter BK, Mangalea MR, Willcox S, Sabet S, Nagoulat MN, Griffith JG, Ventosa A, Oren A, Ma Y (2011). Haloviruses of great salt Lake: a model for understanding viral diversity. Halophiles and hypersaline environments: current research and future trends.

[CR14] Behle WH (1984). In memoriam: Seville flowers (1900-1968). The Great Basin Naturalist.

[CR15] Behrens P, Gwynn JW (1980). Industrial processing of Great Salt Lake brines by Great Salt Lake minerals and chemicals corporation. Great salt Lake: a scientific, historical and economic overview.

[CR16] Bellrose FC (1980). Ducks, geese and swans of North America.

[CR17] Belovsky GE, Stephens D, Perschon C, Birdsey P, Paul D, Naftz D, Baskin R, Larson C, Mellison C, Luft J, Mosley R (2011). The Great Salt Lake ecosystem: long term data and a structural equation approach. Ecosphere.

[CR18] Bingham CP, Gwynn JW (1980). Solar production of potash from the brines of the Bonneville salt flats. Great Salt Lake: a scientific, historical and economic overview.

[CR19] Boyd ES, Hamilton TL, Swanson KD, Howells AE, Baxter BK, Meuser JE, Posewitz MC, Peters JW (2014). [FeFe]-hydrogenase abundance and diversity along a vertical redox gradient in Great Salt Lake, USA. Int J Mol Sci.

[CR20] Boyd ES, Yu R-Q, Barkay T, Hamilton TL, Baxter BK, Naftz DL, Marvin-DiPasquale M (2017). Effect of salinity on mercury methylating benthic microbes and their activities in Great Salt Lake, Utah. Sci Total Environ.

[CR21] Brandt KK, Ingvorsen K (1997). *Desulfobacter halotolerans* sp. Nov., a halotolerant acetate-oxidizing sulfate-reducing bacterium isolated from sediments of Great Salt Lake, Utah. Syst Appl Microbiol.

[CR22] Browne WW (1922). Halophilic bacteria. Proc Soc Exp Biol Med.

[CR23] Cannon JS, Cannon MA, Gwynn JW (2002). The Southern Pacific Railroad trestle—past and present. Great Salt Lake: An Overview of Change.

[CR24] Chidsey TC, Vanden Berg MD, Eby DE (2015). Petrography and characterization of microbial carbonates and associated facies from modern Great Salt Lake and Uinta basins Eocene Green River formation in Utah, USA. Microbial Carbonates in Space and Time: Implications for Global Exploration and Production.

[CR25] Cockerell TDA (1920). Biographical memoir of Alpheus Spring Packard, 1839–1905.

[CR26] Cohenour RE, Thompson KC (1966). Geologic setting of Great Salt Lake, Utah.

[CR27] Collins N (1980). Population ecology of *Ephydra cinerea* Jones (*Diptera: Ephydridae*), the only benthic metazoan of the Great Salt Lake, U.S.. Hydrobiologia.

[CR28] Coltrain JB, Leavitt SW (2002). Climate and diet in Fremont prehistory: economic variability and abandonment of maize agriculture in the Great Salt Lake Basin. Am Antiq.

[CR29] Crisp F, Bell FJ, Bennett AW, & Hebb RG (1880) Transactions of the Society. Journal of the Royal Microscopical Society III(2):560–1120, Williams and Norgate, London

[CR30] Cronin EA, Post FJ (1977). Report of a dematiaceous hyphomycete from the Great Salt Lake. Mycologia.

[CR31] Cuch FS (2000). History of Utah's American Indians.

[CR32] D'Adamo S, Jinkerson RE, Boyd ES, Brown SL, Baxter BK, Peters JW, Posewitz M (2014). Evolutionary and biotechnological implications of robust hydrogenase activity in halophilic strains of *Tetraselmis*. PLoS One.

[CR33] Dainels [s D]LL (1917). On the Flora of Great Salt Lake. Am Nat.

[CR34] Daines LL (1910) Physiological Experiments on some algae of Great Salt Lake. M.S. thesis, University of Utah: QK3.5 1910 D34

[CR35] Defa DR (1994) Lake Bonneville. In: Utah History Encyclopedia. https://heritage.utah.gov/tag/.great-basin. Accessed 22 March 2018

[CR36] Evans FR (1958). Culture of protozoa from Great Salt Lake. The Journal Protozoology.

[CR37] Evans FR (1960). Studies on growth of protozoa from the Great Salt Lake with special reference to *Cristigera* sp. The Journal Protozoology.

[CR38] Evans FR, Thompson JC (1964). *Pseudocohnilembidae* n. fam., a hymenostome ciliate family containing one genus, *Pseudocohnilembus* ng, with three new species. The Journal of Protozoology.

[CR39] Felix EA, Rushforth SR, Greer D (1977). The algal flora of the Great Salt Lake, Utah: a preliminary report. Desertic Terminal Lakes.

[CR40] Felix EA, Rushforth SR (1979). The algal flora of the Great-Salt-Lake, Utah, USA. Nova Hedwigia.

[CR41] Felix EA, Rushforth SR (1980). Biology of the south arm of the Great Salt Lake, Utah. Utah Geology Mineral Survey Bulletin, Salt Lake City.

[CR42] Flowers S (1934). Vegetation of the Great Salt Lake region. Bot Gaz.

[CR43] Flowers S (1957). Ethnobryology of the Goshute Indians of Utah. Bryologist.

[CR44] Flowers S, Evans FK, Boyko H (1966). The flora and fauna of the Great Salt Lake region, Utah. Salinity and aridity, new approaches to old problems.

[CR45] Fraser RS, Argall CI (1954). Survival of *E. coli* in water from Great Salt Lake. Sewage and Industrial Wastes.

[CR46] Frederick E (1924) On the bacterial flora of Great Salt Lake and the viability of other microorganisms in Great Salt Lake water. M.S. thesis, University of Utah

[CR47] Frémont JC (1845). Report of the exploring expedition to the Rocky Mountains in the year 1842 and to Oregon and North California in the years 1843–44: printed by order of the Senate of the United States.

[CR48] FRIENDS of Great Salt Lake (2018) Doyle Stephens scholarship. In: FRIENDS of Great Salt Lake. https://fogsl.org/programs/doyle-stephens-scholarship. Accessed 28 March 2018

[CR49] Great Salt Lake Artemia Association (2018) http://www.gsla.us/. Accessed 28 March 2018

[CR50] Greer DC (1971). Annals map supplement fourteen: great salt Lake, Utah. Ann Assoc Am Geogr.

[CR51] Harvard University. https://huh.harvard.edu/pages/farlow-herbarium-fh. Accessed 6 March 2018

[CR52] Hayden FV (1873). Sixth annual report of the United States Geological Survey of the Territories, embracing portions of Montana, Idaho, Wyoming, and Utah; being a report of progress of the explorations for the year 1872.

[CR53] Horsfield M (2016) The enduring legacy of Josephine Tilden. In: Hakai Magazine https://www.hakaimagazine.com/article-long/enduring-legacy-josephine-tilden. Accessed 10 March 2018

[CR54] Hunter MR (1943). Utah in her western setting.

[CR55] Jones DT (1944). Two protozoans from Great Salt Lake. Bulletin of the University of Utah.

[CR56] Jones DL, Baxter BK (2016). Bipyrimidine signatures as a photoprotective genome strategy in G+ C-rich halophilic archaea. Life.

[CR57] Jones DL, Baxter BK (2017). DNA repair and photoprotection: mechanisms of overcoming environmental ultraviolet radiation exposure in halophilic archaea. Front Microbiol.

[CR58] Jones BF, Naftz DL, Spencer RJ, Oviatt CG (2009). Geochemical evolution of Great Salt Lake, Utah, USA. Aquat Geochem.

[CR59] Keck W, Hassibe W (1979). The Great Salt Lake.

[CR60] Kirkpatrick R (1934) The life of Great Salt Lake, with special reference to the algae. M.S. thesis, University of Utah: QK3.5 1934 K57

[CR61] Lambourne A (1909). Our Inland Sea.

[CR62] Larsen H (1986). Halophilic and halotolerant microorganisms-an overview and historical perspective. FEMS Microbiol Lett.

[CR63] Larson CA, Belovsky GE (2013). Salinity and nutrients influence species richness and evenness of phytoplankton communities in microcosm experiments from Great Salt Lake, Utah, USA. J Plankton Res.

[CR64] Lindsay MR, Anderson C, Fox N, Scofield G, Allen J, Anderson E, Bueter L, Poudel S, Sutherland K, Munson-McGee JH, Van Nostrand JD, Zhou J, Spear JR, Baxter BK, Lageson DR, Boyd ES (2017). Microbialite response to an anthropogenic salinity gradient in Great Salt Lake, Utah. Geobiology.

[CR65] Macroalgal Herbarium Portal. http://macroalgae.org/portal/collections/exsiccati/index.php?ometid=5. Accessed 3 March 2018

[CR66] Madison RJ (1970) Effects of a causeway on the chemistry of the brine in Great Salt Lake, Utah. In: Utah Geological and Mineralogical Survey Water-Resources Bulletin 14

[CR67] Madsen DB (1989). A grasshopper in every pot: in the desert west, small game makes good sense. Nat Hist.

[CR68] Madsen DB, Beck C (1999). The nature of Great Basin environmental change during the Pleistocene/Holocene transition and its possible impact on human populations. Models for the millennium: the current status of Great Basin anthropological research.

[CR69] Madsen DB, Parezoand NJ, Janetski JC (2014). Eight decades eating dust: a short history of archaeological research at danger cave. Archaeology for all times: papers in honor of don D. Fowler.

[CR70] Madsen DB (2015). A framework for the initial occupation of the Americas. PaleoAmerica.

[CR71] Madsen DB, Oviatt CG, Shroder JF (2016). The early human occupation of the Bonneville Basin. Lake Bonneville: a scientific update.

[CR72] Madsen DB, Kirkman J (1988). Hunting hoppers. Am Antiq.

[CR73] Marcarelli AM, Wurtsbaugh WA, Griset O (2006). Salinity controls phytoplankton response to nutrient enrichment in the Great Salt Lake, Utah, USA. Can J Fish Aquat Sci.

[CR74] May SO (1978) The effect of various environmental factors on the growth of a red pigmented *Dunaliella* species from the Great Salt Lake. M.S. thesis, Utah State University: 3381

[CR75] Meuser JE, Baxter BK, Spear JR, Peters JW, Posewitz MC, Boyd ES (2013). Contrasting patterns of community assembly in the stratified water column of Great Salt Lake, Utah. Microb Ecol.

[CR76] Miller DE (1948). John C. Frémont in the Great Salt Lake region. Historian.

[CR77] Miller DE (1969). Great Salt Lake past and present.

[CR78] Moore HF (1899) The feasibility of introducing useful marine animals into the waters of Great Salt Lake. US Fish Commission Report, pp 229–250

[CR79] Morgan DL, Quaife MM (1947). The Great Salt Lake. The American Lakes series.

[CR80] Motlagh AM, Bhattacharjee AS, Coutinho FH, Dutilh BE, Casjens SR, Goel RK (2017) Insights of phage-host interaction in hypersaline ecosystem through metagenomics analyses. Front Microbiol 8. 10.3389/fmicb 10.3389/fmicb.2017.00352 PMC5334351 28316597

[CR81] Muir J (1877) Letter dated "Lake point, Utah, may 20, 1877." Sierra Club. http://vault.sierraclub.org/john_muir_exhibit/writings/steep_trails/chapter_8.aspx. Accessed 6 March 2018

[CR82] Naftz DL, Millero FJ, Jones BF, Green WR (2011). An equation of state for hypersaline water in Great Salt Lake, Utah, USA. Aquat Geochem.

[CR83] Neill J, Leite B, Gonzales J, Sanchez K & Luft J (2016) 2015 Great Salt Lake Eared Grebe aerial photo survey. Annual Report Great Salt Lake Ecosystem Program. Utah Division of Wildlife Resources

[CR84] Oren A (1999). Microbiological studies in the Dead Sea: future challenges toward the understanding of life at the limit of salt concentrations. Hydrobiologia.

[CR85] Oring LW, Neel L & Oring KE (2000) Intermountain West Regional Shorebird Plan Version 1.0. In: U.S. Shorebird Conservation Plan. https://www.shorebirdplan.org/wp-content/uploads/2013/01/IMWEST4.pdf. Accessed 18 March 2018

[CR86] Oviatt CG, Thompson RS, Kaufman DS, Bright J, Forester RM (1999). Reinterpretation of the Burmester Core, Bonneville Basin, Utah. Quat Res.

[CR87] Oviatt CG, Madsen DB, Schmitt DN (2003). Late Pleistocene and early Holocene rivers and wetlands in the Bonneville basin of western North America. Quat Res.

[CR88] Oviatt CG, Madsen DM, Miller DM, Thompson RS, McGeehin JP (2015). Early Holocene Great Salt Lake, USA. Quat Res.

[CR89] Pack D (1919). Two Ciliata of Great Salt Lake. Biol Bull.

[CR90] Packard AS (1871). On insects inhabiting salt water. Am J Sci.

[CR91] Packard AS (1879). The sea-weeds of Salt Lake. Am Nat.

[CR92] Parnell JJ, Crowl TA, Weimer BC, Pfrender ME (2009). Bio-diversity in microbial communities: system scale patterns and mechanisms. Mol Ecol.

[CR93] Parnell JJ, Rompato G, Latta LC, Pfrender ME, Van Nostrand JD, He Z, Zhou J, Andersen G, Champine P, Ganesan B, Weimer BC (2010). Functional biogeography as evidence of gene transfer in hypersaline microbial communities. PLoS One.

[CR94] Parnell JJ, Rompato G, Crowl TA, Weimer BC, Pfrender ME (2011). Phylogenetic distance in Great Salt Lake microbial communities. Aquat Microb Ecol.

[CR95] Parr RL, Carlyle SW, O'Rourke DH (1996). Ancient DNA analysis of Fremont Amerindians of the Great Salt Lake wetlands. Am J Phys Anthropol.

[CR96] Patrick R (1936). Some diatoms of Great Salt Lake. Bulletin of the Torrey Botanical Club.

[CR97] Paul DS & Manning AE (2016) Great Salt Lake Waterbird survey five-year report 1997–2001. In: Great Salt Lake Ecosystem Program and Utah Division of Wildlife Resources. https://www.wildlife.utah.gov/gsl/waterbirdsurvey/. Accessed 20 March 2018

[CR98] Perl SM, Vaishampayan PA, Corsetti FA, Piazza O, Ah-med M, Willis P, Creamer JS, Williford KW, Flannery DT, Tuite ML, Ehlmann BL, Bhartia R, Baxter BK, Butler JK, Hodyss R, Berelson WM, Nealson KH (2016). Identification and validation of bigenic preservation: defining constraints within Martian mineralogy.

[CR99] Post FJ (1975). Life in the Great Salt Lake. Utah Science.

[CR100] Post FJ (1977). The microbial ecology of the Great Salt Lake. Microb Ecol.

[CR101] Post FJ, Gwynn JW (1980). Biology of the north arm. Great Salt Lake: a scientific, historical and economic overview.

[CR102] Post FJ (1980). Oxygen-rich gas domes of microbial origin in the salt crust of the Great Salt Lake, Utah. Geomicrobiol J.

[CR103] Post FJ (1981). Microbiology of the Great Salt Lake north arm. Hydrobiologia.

[CR104] Post FJ, Stube JC (1988). A microcosm study of nitrogen utilization in the Great Salt Lake, Utah. Hydrobiolgia.

[CR105] Post FJ, Borowitzka LJ, Borowitzka MA, Mackay B, Moulton T (1983). The protozoa of a western Australian hypersaline lagoon. Hydrobiologia.

[CR106] Pugin B, Blamey JM, Baxter BK, Wiegel J (2012). *Amphibacillus cookii* sp. nov., a facultatively aerobic, spore-forming, moderately halophilic, alkalithermotolerant bacterium. Int J Syst Evol Microbiol.

[CR107] Raghavan M, Steinrücken M, Harris K, Schiffels S, Rasmussen S, DeGiorgio M, Albrechtsen A, Valdiosera C, Ávila-Arcos MC, Malaspinas AS, Eriksson A (2015). Genomic evidence for the Pleistocene and recent population history of Native Americans. Science.

[CR108] Rarick E (2008). Desperate passage: the Donner Party's perilous journey west.

[CR109] Reddy YJR (1971) A Description of Morphology of a New Species of *Euplotes* from Great Salt Lake, Utah. M.S. thesis, University of Utah: QL 3.5 1972 R4

[CR110] Roberts AJ (2013). Avian diets in a saline ecosystem: great salt Lake, Utah, USA. Human–Wildlife Interactions.

[CR111] Rothpletz A (1892) Report on *Gleothece* and *Gleocystis*. Botanik. Centr, p 35

[CR112] Rushforth SR, Felix AF (1982). Biotic adjustments to changing salinities in the Great Salt Lake, Utah, USA. Microb Ecol.

[CR113] Rushforth SR & Merkley GS (1988) Comprehensive list by habitat of the algae of Utah, USA. The Great Basin Naturalist, pp 154–179

[CR114] Scientific American (1861) Great Salt Lake 9:131–132

[CR115] Shen PS, Domek MJ, Sanz-García E, Makaju A, Taylor RM, Hoggan R, Culumber MD, Oberg CJ, Breakwell DJ, Prince JT, Belnap DM (2012). Sequence and structural characterization of Great Salt Lake bacteriophage CW02, a member of the T7-like supergroup. J Virol.

[CR116] Shroder JF, Cornwell K, Oviatt CG, Lowndes TC, Oviatt CG, Shroder JF (2016). Landslides, alluvial fans, and dam failure at Red Rock Pass: the outlet of Lake Bonneville. Lake Bonneville a scientific update.

[CR117] Smith WW (1936) Evidence of a bacterial flora indigenous to the Great Salt Lake. M.S. thesis, University of Utah

[CR118] Spencer H (1904). The progress of science. Popular Science Monthly.

[CR119] Stansbury H (1855). Exploration of the valley of the Great Salt Lake: including a reconnaissance of a new route through the Rocky Mountains.

[CR120] State of Utah (2018a) https://wildlife.utah.gov/gsl/industries/index.php. Accessed 18 March 2018

[CR121] State of Utah (2018b) https://wildlife.utah.gov/habitat/farmington_bay.php. Accessed 18 March 2018

[CR122] State of Utah (2018c) https://wildlife.utah.gov/gsl/. Accessed 28 March 2018

[CR123] Stephens DW (1974). A summary of biological investigations concerning the Great Salt Lake, Utah (1861–1973). Great Basin Naturalist.

[CR124] Stephens DW (1990). Changes in lake levels, salinity and the biological community of Great Salt Lake, 1847–1987. Developments in. Hydrobiology.

[CR125] Stephens DW, Pitman J, Carroll A (1998). Salinity-induced changes in the aquatic ecosystems of Great Salt Lake, Utah. Modern and ancient Lake systems.

[CR126] Stephens DW & Gillespie DM (1972) Community structure and ecosystem analysis of the Great Salt Lake. In: Riley JP (ed) The Great Salt Lake and Utah's water resources, Proceedings First Annual Conference of Utah Section American Water Resources Association. Utah Water Research Lab, Utah State University, Logan, pp 66–72

[CR127] Stephens DW, Gillespie DM (1976). Phytoplankton production in the Great Salt Lake, Utah, and a laboratory study of algal response to enrichment. Limnol Oceanogr.

[CR128] Talmage JE (1889). The waters of the Great Salt Lake. Science.

[CR129] Talmage JE (1900). The Great Salt Lake-present and past.

[CR130] Tazi L, Breakwell DP, Harker AR, Crandall KA (2014). Life in extreme environments: microbial diversity in Great Salt Lake, Utah. Extremophiles.

[CR131] The Church of Jesus Christ of Latter-Day Saints (1997). On the trail in July.

[CR132] The Deseret News (1907) Weary path trodden by intrepid band, to the Shores of America’s Dead Sea pp 4–5, July 27, 1907

[CR133] Salt Lake Tribune (1931) Regal coed: U engineers elect queen. April 7, 1931

[CR134] Tilden JE (1898) American algae. Century III: 298

[CR135] Tilden JE (1910). Minnesota algae: the Myxophyceae of North America and adjacent regions including central America, Greenland, Bermuda, the West Indies and Hawaii.

[CR136] United States Bureau of Reclamation (1962). Bear River project, part I, feasibility report, Oneida division, Idaho and Utah. Part II, reconnaissance report, blacksmith fork division, Utah, 86.

[CR137] United States Division of Fish and Wildlife (2018) https://www.fws.gov/Refuge/Bear_River_Migratory_Bird_Refuge/about.html. Accessed 18 March 2018

[CR138] United States Geologic Survey (2018) http://ut.water.usgs.gov/greatsaltlake/elevations Accessed 18 March 2018

[CR139] University of Utah (2018) http://medicine.utah.edu/pathology/microbiology-immunology. Accessed 21 March 2018

[CR140] Verrill AE (1869) Territories of Wyoming and Idaho: U.S. Geological & Geographical Survey Annual Report 12 (1878), Pt 1. U.S. Government Printing Office, Washington, DC

[CR141] Vorhies CT (1917). Notes on the fauna of the Great Salt Lake. Am Nat.

[CR142] Weimer BC, Rompato G, Parnell J, Gann R, Ganesan B, Navas C, Gonzalez M, Clavel M, Albee-Scott S (2009). Microbial biodiversity of Great Salt Lake, Utah. Natural Resources and Environmental Issues.

[CR143] Westminster College (2018) www.greatsaltlakeinstitute.org. Accessed 7 March 2018

[CR144] Wintrobe MM (1982). Medical education in Utah. West J Med.

[CR145] Woese CR, Fox GE (1977). Phylogenetic structure of the prokaryotic domain: the primary kingdoms. Proc Natl Acad Sci U S A.

[CR146] Wotipka S (2014) Brine shrimp by the billions in the Great Salt Lake. High Country News. https://www.hcn.org/issues/46.10/brine-shrimp-by-the-billions-in-the-great-salt-lake

[CR147] Wurtsbaugh WA, Gliwicz ZM (2001). Limnological control of brine shrimp population dynamics and cyst production in the Great Salt Lake, Utah. Hydrobiologia.

[CR148] Wurtsbaugh WA, Gardberg J, Izdepski C (2011). Biostrome communities and mercury and selenium bioaccumulation in the Great Salt Lake (Utah, USA). Sci Total Environ.

[CR149] Wurtsbaugh WA, Miller C, Null SE, DeRose RJ, Wilcock P, Hahnenberger M, Howe F, Moore J (2017). Decline of the world's saline lakes. Nat Geosci.

[CR150] Zobell CE (1937). Direct microscopic evidence of an autochthonous bacterial flora in Great Salt Lake. Ecology.

[CR151] Zobell CE, Anderson DQ, Smith WW (1937). The bacteriostatic and bactericidal action of great salt Lake water. J Bacteriol.

